# 3D Reconstruction with Single-Shot Structured Light RGB Line Pattern

**DOI:** 10.3390/s21144819

**Published:** 2021-07-14

**Authors:** Yikang Li, Zhenzhou Wang

**Affiliations:** College of Electrical and Electronic Engineering, Shandong University of Technology, Zibo 255000, China; 14111103010@stumail.sdut.edu.cn

**Keywords:** 3D reconstruction, single shot, slope difference distribution, line indexing, structured light, phase map

## Abstract

Single-shot 3D reconstruction technique is very important for measuring moving and deforming objects. After many decades of study, a great number of interesting single-shot techniques have been proposed, yet the problem remains open. In this paper, a new approach is proposed to reconstruct deforming and moving objects with the structured light RGB line pattern. The structured light RGB line pattern is coded using parallel red, green, and blue lines with equal intervals to facilitate line segmentation and line indexing. A slope difference distribution (SDD)-based image segmentation method is proposed to segment the lines robustly in the HSV color space. A method of exclusion is proposed to index the red lines, the green lines, and the blue lines respectively and robustly. The indexed lines in different colors are fused to obtain a phase map for 3D depth calculation. The quantitative accuracies of measuring a calibration grid and a ball achieved by the proposed approach are 0.46 and 0.24 mm, respectively, which are significantly lower than those achieved by the compared state-of-the-art single-shot techniques.

## 1. Introduction

Non-contact optical surface 3D imaging technology has made tremendous progress in the last decades. Traditional methods are challenged by more flexible alternatives [1]. Nevertheless, robust 3D reconstruction of moving and deforming objects remains challenging. To reconstruct a dynamic scene, the optimum choice is the single-shot method, and state-of-the-art single-shot methods can be divided into four categories. The first category is time of flight (TOF), the second category is passive stereo vision (PSV), the third category is active stereo vision (ASV), and the fourth category is structured light (SL). Each category has its representative product. For instance, Kinect V2 is the representative product of the first category. Despite its wide use and great popularity, its performance is not stable. For instance, it is reported that Kinect V2 is robust in reconstructing rigid scenes while it fails in providing a reasonable reconstruction result on non-rigidly deforming scenes [2]. The representative product of both PSV and ASV is Intel D435. The passive mode of Intel D435 performs poorly while measuring objects that lack textures [3]. The active mode of Intel D435 adopts an infrared radiation (IR) laser projector to generate the laser speckle patterns [4]. It performs better than other methods for face identification and face expression recognition as reported by Luca et al. [5]. The accuracies of ASV and SL are higher than those of TOF and PSV. In addition, the cost of SL is lower than that of ASV. Therefore, SL has been favored for reconstructing dynamic scenes for many decades, and a great number of single-shot SL methods have been proposed based on the different types of the coded patterns, as reviewed by Wang [1].

Fourier transform profilometry (FTP) [6] is one of the most popular single-shot structured light methods. It decodes a single high-frequency sinusoidal pattern in the frequency domain to calculate the phase map and the depth information. The carrier frequency needs to be designed as high as possible to reduce the aliasing error. A band-pass filter is used to obtain the modulated phase in the frequency domain. The drawbacks of FTP include (1) sensitivity to noise, light interference, and the optical properties of the measured objects; (2) requirement of a non-trivial phase unwrapping process; (3) low accuracy in measuring complex surfaces. To overcome the drawbacks of FTP, the structured light line pattern profilometry (LPP) was proposed to reconstruct the 3D profile by single-shot in [7]. Different from FTP, LPP calculates the depth information solely in the spatial domain. LPP extracts the distorted line pattern with a series of image processing methods and then indexes the distorted lines uniquely. Because the extracted lines instead of the full-resolution plane are used, LPP is more resistant to noise and light interference. A typical example of the high resistance to noise and light interference can be found in [8] where the laser lines are extracted robustly under extremely strong arc light while measuring the geometry of the weld pool. Because of advances in the image segmentation method, the lines can be extracted robustly while measuring the objects with different optical properties, as shown by Wang et al. [8]. In addition, the phase map calculated by LPP is not wrapped and thus does not require the phase unwrapping process. To measure complex surfaces, more subtle line clustering methods are required by LPP. For instance, a specific line clustering method was proposed to reconstruct the deforming human face in [9], and the line clustering method is designed based on the characteristics of the human face. Consequently, it cannot be used for measuring the 3D shapes of other complex deforming objects, although it performed better than other methods [10,11,12,13,14] in 3D face reconstruction.

In this paper, we propose an exclusion-based line clustering method that can be used for measuring any type of deforming shape. To facilitate the clustering process, we designed the lines in red, green, and blue respectively instead of using a single white color [8,9,15]. The lines in different colors are separated from each other at equal intervals. The minimum interval between the lines in the same color is three times as large as the minimum interval between the adjacent lines in the coded pattern. Therefore, the lines in the same color have little intersection after they are distorted by depth because of their large intervals. After the projected RGB line pattern is captured by a color charge coupled device (CCD) camera, the lines in red, green, and blue are segmented in the hue saturation value (HSV) color space. The segmented red lines, green lines, and blue lines are clustered independently with the proposed line clustering method. To verify the effectiveness of the proposed approach, we conducted both quantitative and qualitative experiments that included reconstructing the static objects for 3D reconstruction accuracy evaluation and reconstructing the moving and deforming object for the line clustering (indexing) robustness evaluation.

In summary, the major contributions of this paper include:

(1) A fully automatic color stripe segmentation method based on SDD is proposed to extract the RGB line pattern robustly. State-of-the-art methods rely on manually specified thresholds obtained by trial-and-error analysis or the classic classification methods to segment the color stripes. The trial-and-error analysis method is time-consuming and cannot guarantee that the optimal thresholds are always selected for each image. In addition, the demonstration in the paper shows that none of the compared classic classification methods can separate all the adjacent stripes from each other robustly. Due to the bottleneck problem of image segmentation, there are no 3D surface imaging products that are based on the segmented structured light patterns yet, even though the related theory and idea had been proposed for more than three decades. The proposed SDD segmentation method has the potential to solve this bottleneck problem for the single-shot dot-pattern- or line-pattern-based structured light methods.
(2) An exclusion-based line clustering method is proposed to index the segmented stripes robustly. State-of-the-art methods have included different ways to index the segmented lines or dots uniquely. However, they may fail when complex and deforming objects are reconstructed. In [7], a line clustering method was proposed to index the lines from top to bottom, but it is only robust for simple and rigid objects. In [8,9], the line-slope-based clustering method was proposed to index the lines distorted by complex and deforming objects. However, its accuracy is easily affected by occlusion and large discontinuity. The line clustering method proposed in this paper is more robust than the previous methods [7,8,9] in indexing the lines distorted by complex and deforming objects, which is validated by the 3D reconstruction results of a deforming face and a deforming hand in the attached videos.
(3) An extensive comparison of the proposed method with state-of-the-art single-shot 3D surface imaging methods (products) was conducted by reconstructing the deforming face and the deforming hand continuously, which is a necessary condition to verify the effectiveness of a single-shot structured light method.

## 2. Related Work

The structured light 3D imaging technique reconstructs the sampled points on the surface of the measured object based on different working principles. Based on the sampling resolution, state-of-the-art structured light methods can be divided into three categories: (1) dot-pattern-based method [15,16,17,18,19,20,21,22,23,24]; (2) line (stripe)-pattern-based method [7,8,9,25,26,27,28,29,30], and (3) plane-pattern-based method [6,31,32]. The dot-pattern-based methods sample the surface of the object sparsely with the specifically coded dot patterns. Image segmentation [16,17,18,19,20] or feature detection [21,22,23,24] are used to extract the dot pattern from the acquired pattern image. Each extracted dot is then indexed uniquely for 3D depth calculation with different principles. The line-pattern-based methods sample the surface of the object significantly more densely than the dot-pattern-based methods. Similarly, image segmentation [26,29,30] or feature detection [25,27,28] can be used to extract the line pattern from the acquired pattern image. The plane-pattern-based methods sample the surface of the object with the resolution of the projector. The correspondence between the camera and the projector is pixel to pixel. To distinguish the pixels from each other, the pixels in the coded plane pattern vary according to the function of a sine wave. Unlike the dot pattern and the line pattern, the plane pattern is not identifiable spatially within a single image. As a result, image segmentation or feature detection cannot extract the phase map from a single image solely based on the spatial information. Currently, there are two popular ways for phase map calculation. One is FTP [6] and the other is phase-shifting profilometry (PSP) [30,31]. FTP calculates the phase map in the frequency domain by selecting the fundamental component of the Fourier transformed pattern image with a band-pass filter. To reduce the aliasing error, the carrier frequency of FTP is required to be as high as possible. In reality, alias is unavoidable for FTP to measure the non-planar objects, and consequently the phase map generated by FTP is usually not accurate enough for depth calculation. PSP calculates the phase map in the temporal domain by solving the united equation of the acquired pattern images with different phase-shifting values. In theory, PSP yields an exact analytical solution of the phase map. The major drawback of PSP is that it requires the object to be static when projecting multiple plane patterns onto it. Consequently, PSP is not suitable for measuring deforming and moving objects by nature. To reconstruct deforming and moving objects, the dot pattern or line pattern is usually preferred and used for the structured light method development.

One major challenge of dot-pattern- or line-pattern-based structured light methods is to extract the dots or the lines automatically and robustly. Since the color dots or the color lines are designed distinctively, threshold selection or color classification should be able to segment them robustly. Yet few works have described in detail how the dots or lines were segmented. In [17], the valleys and peaks of the histogram were analyzed and a threshold was selected manually based on the trial-and-error analysis. Obviously, the manually selected threshold cannot always guarantee that the dots or lines are segmented optimally, especially while reconstructing complex deforming objects. In [19], the authors demonstrated that the reconstruction was incomplete because of the challenge of color dot segmentation. In [26], the authors mentioned that the stripes could be segmented by color classification or edge detection, and they basically advocated color classification. Unfortunately, they did not disclose what color classification method was used by them. In [27,28], the authors utilized the local maxima to segment the stripes. In reality, the captured images are usually affected by color crosstalk, specular reflection, and light interference. As a result, the local maxima-based method could not segment the stripes as accurately as the threshold selection methods or the classification methods. In [29], the authors specified the thresholds for the red, green, and blue stripes as 125, 90, and 95, respectively. Together with the comparison of the gray levels in different channels, the stripes could be segmented effectively. However, the segmentation accuracy was affected by the leakage in the color separation filters and poor color alignment. To our best knowledge, it is difficult to segment the color lines solely based on single-threshold selection or color classification. In this paper, we will address this challenge by proposing a robust RGB line pattern segmentation method based on SDD.

The second challenge of dot-pattern- or line-pattern-based structured light methods is to index the extracted dots or lines uniquely and robustly. Although the stripe indexing issue has been tackled for many decades, it has not yet been solved for the following two reasons: (1) there are no line (stripe) indexing-based single-shot 3D imaging products yet, (2) the proposed line (stripe) indexing methods have not yet been evaluated with deforming and moving objects extensively. Consequently, the robustness of the proposed methods cannot be verified. For instance, only one reconstruction result of a face was demonstrated in [27,28]. Eight reconstruction results of a deforming face were demonstrated in [26], and 18 reconstruction results of a deforming hand were demonstrated in [30]. There are even some single-shot structured light methods that have not been tested with deforming and moving objects at all. In this paper, we will validate the proposed method extensively with the deforming hand and the deforming face. In addition, we extensively compare the 3D reconstruction results of the moving face and hand by the proposed method with those reconstructed by state-of-the-art products in the attached videos.

Although the line (stripe) indexing problem remains unresolved, many interesting and promising line indexing methods have been proposed in the past decades. For instance, the flood fill algorithm was proposed by Robinson et al. to index the stripes in [27]. A maximum spanning tree-based algorithm was proposed by Brink et al. to index the stripes in [28]. The maximum spanning tree of a graph was used to define potential connectivity and adjacency in stripes. Despite a significant improvement in accuracy over existing methods, e.g., the flood fill-based method [27], the maximum spanning tree algorithm could not address the discontinuity in surface depth effectively. A survival of the fittest criterion was proposed by Boyer and Kak to index the stripes uniquely in [29]. The stripe indexing was treated as a labeling problem and formulated as an energy minimization problem by Koninckx and Van Gool in [30]. To solve the minimization problem, a weighted least squares solution was proposed to cast the problem into the search for a minimal cut on a graph. Though the theory sounds good, the reconstructed deforming hands had serious flaws, e.g., the black holes in the reconstructed palm. In this paper, we will address this challenge by proposing a robust line clustering method to index the stripes based on exclusion. With the assumption that the reconstructed surface is continuous and smooth, a line model is computed for each clustered line to make the reconstructed surface continuous and smooth. As a result, the occlusion problem, the discontinuity problem, and other similar problems, e.g., the black hole problem as shown in [30], can be solved effectively.

The major differences between the proposed method and the existing methods [26,27,28,29,30] are summarized in Table 1. Compared to the proposed method, the major weaknesses of the existing methods include: (1) the existing methods were not able to segment the lines (stripes) as robustly as the proposed method was able to; (2) the existing methods were not able to index the lines (stripes) as robustly as the proposed method was able to. Consequently, their results in reconstructing deforming and moving objects are inferior to those of the proposed method, which can be verified by comparing the reconstruction results of the deforming hand in this paper and those shown in [30].

## 3. The Proposed Approach

**Figure 1:**
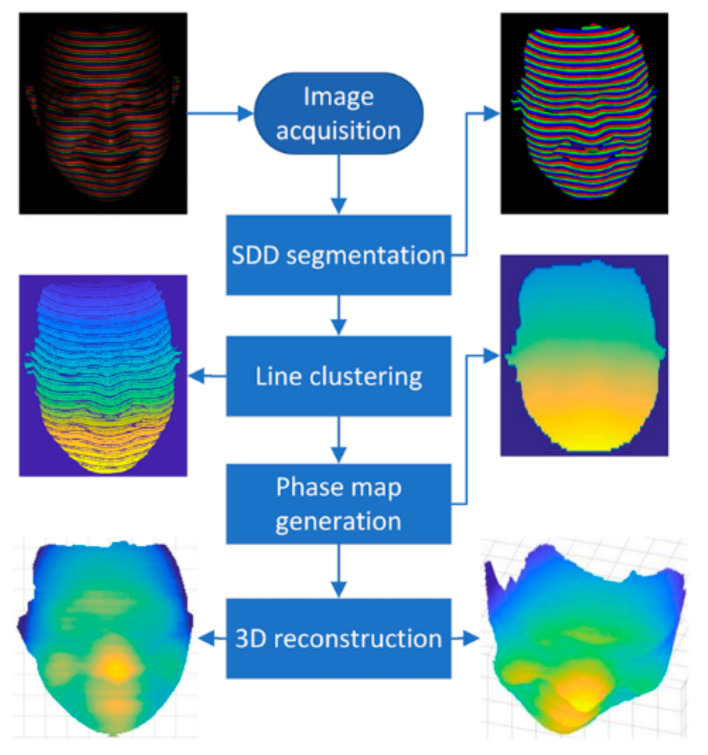
The flowchart of the proposed approach.

The flowchart of the proposed approach is shown in Figure 1. Firstly, the acquired image is segmented in the HSV color space by the SDD image segmentation method [33,34]. The segmented lines in different colors are then indexed by the proposed exclusion-based line clustering methods. The indexed lines in different colors are fused together to generate the final indexing lines. The phase map is computed from the final indexed lines and the 3D shape is reconstructed with the phase map.

### 3.1. The Proposed Pattern Extraction Method

**Figure 2:**
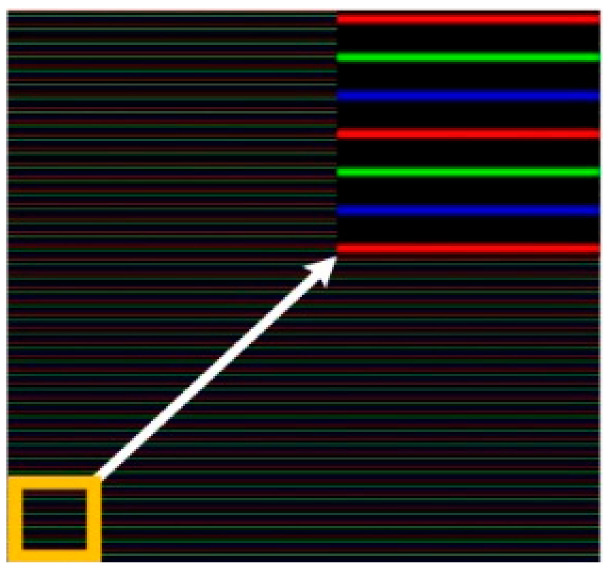
Illustration of the coded pattern.

**Figure 3:**
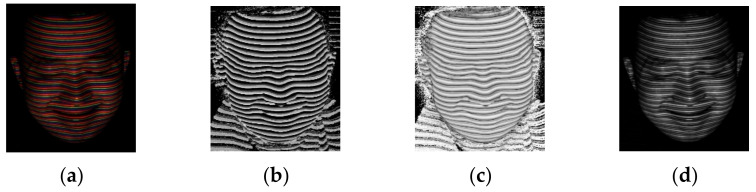
Illustration of the acquired pattern image in H, S, and V channels. (**a**) The acquired RGB pattern image; (**b**) The H channel of the acquired pattern image; (**c**) The S channel of the acquired pattern image; (**d**) The V channel of the acquired pattern image.

The coded RGB pattern is illustrated in Figure 2. The red lines, green lines, and blue lines are in the same intervals, parallel and repeated in cycles to form the RGB pattern. Between the adjacent lines, there are dark parts to separate them from each other. The intensities in different channels of the RGB color space contain both the color information and the lighting information, which makes the color segmentation problematic. On the other hand, color and brightness are separated in different channels as hue and value, which makes the color segmentation straightforward. Therefore, the RGB color space is transformed into the HSV color space for line segmentation. The transformation from the RGB color space to the HSV color space is formulated as follows.

(1) $$V(i,j)=\max\left(R(i,j),G(i,j),B(i,j)\right),$$

(2) $$M(i,j)=\min\left(R(i,j),G(i,j),B(i,j)\right),$$

(3) $$S(i,j)=\frac{V(i,j)-M(i,j)}{V(i,j)},$$

(4) $$H(i,j)=\left\{\begin{array}{l} 60\times \frac{G(i,j)-B(i,j)}{V(i,j)-M(i,j)},R(i,j)=V(i,j)\\ 120+\frac{B(i,j)-R(i,j)}{V(i,j)-M(i,j)},G(i,j)=V(i,j)\\ 240+\frac{R(i,j)-G(i,j)}{V(i,j)-M(i,j)},B(i,j)=V(i,j) \end{array}\right.$$

where (i,j) denotes the index of the pixel in the acquired pattern image. H represents the *H* channel of the pattern image, S represents the *S* channel of the pattern image, and V represents the *V* channel of the pattern image. R represents the *R* channel of the pattern image, G represents the *G* channel of the pattern image, and B represents the *B* channel of the pattern image. From the above HSV color model, it is seen that the coded RGB pattern has the following characteristics: (1) the RGB color is distinguished in the *H* channel, (2) the difference between the RGB line part and the adjacent dark part is highlighted in the *S* channel, (3) the brightness of the RGB line part is highlighted in the *V* channel. The illustrated HSV channels in Figure 3 also correlate with these three characteristics highly. Figure 3a shows the RGB color image acquired by the CCD camera. Figure 3b–d shows the *H* channel, the *S* channel, and the *V* channel of the acquired image, respectively.

**Figure 4:**
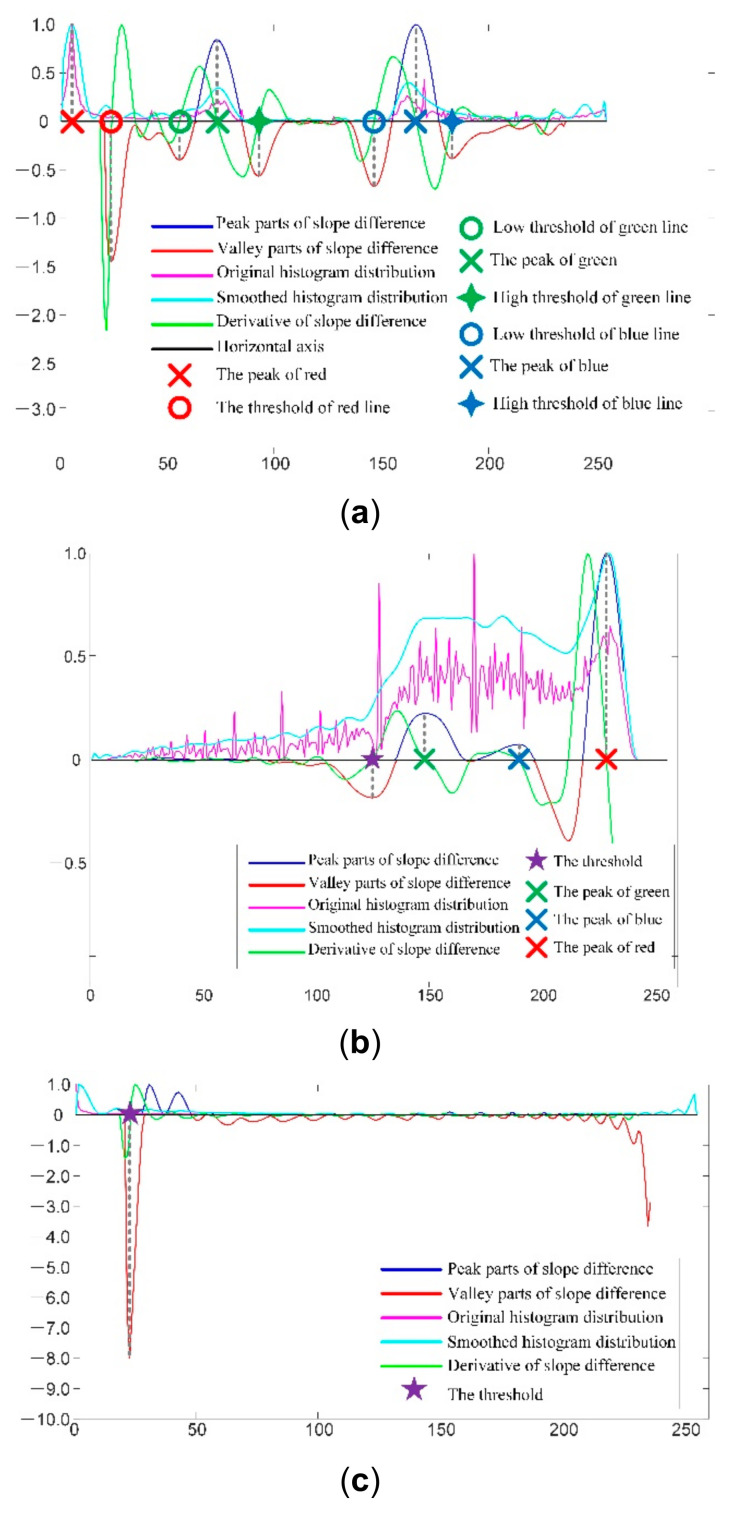
Illustration of the SDD threshold selection process for the acquired pattern image in H, S, and V channels. (**a**) The SDD threshold selection process in the H channel; (**b**) The SDD threshold selection process in the S channel; (**c**) The SDD threshold selection process in the V channel.

The line segmentation method is proposed based on these three characteristics: (1) the lines in red, green, and blue are distinguished in the H channel. Thus, there should be three clustering centers in the histogram of the H channel that correspond to the red pixels, the green pixels, and the blue pixels, respectively. The clustering centers are calculated by SDD as described in [33,34]. For each clustering center, there are two corresponding SDD valleys. One is on its left and the other is on its right. They are selected as the thresholds to segment the lines in the corresponding color. The process of selecting the double thresholds for the red lines, blue lines, and green lines in the H channel by SDD is illustrated in Figure 4a; (2) the RGB line parts and the dark parts are distinguished in the S channel. Thus, there should be one global threshold that can distinguish the RGB line part and the dark part. The global threshold is calculated with SDD as described in [33,34]. The process of the selecting the global threshold to separate the line part from the dark part in the S channel by SDD is illustrated in Figure 4b; (3) because the brightness of the RGB line part is highlighted in the V channel, the region of interest (ROI) of the RGB lines can be obtained from the V channel by a global threshold. The global threshold is calculated with SDD as described in [33,34]. The process of the selecting the global threshold to obtain the ROI of the RGB lines in the S channel with SDD is illustrated in Figure 4c.

**Figure 5:**
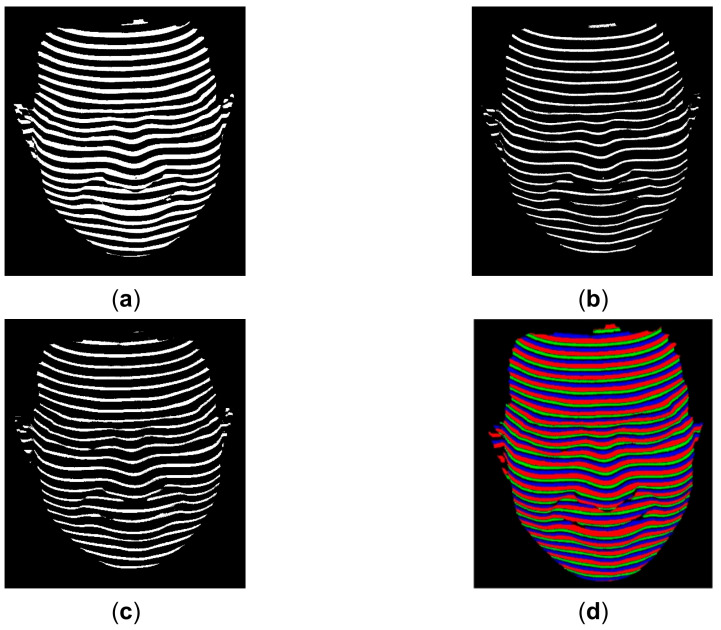
Illustration of the segmented lines in different colors. (**a**) The segmented lines in red; (**b**) The segmented lines in green; (**c**) The segmented lines in blue; (**d**) The combination of the RGB lines.

With all the thresholds computed in the HSV channels, the RGB lines are segmented with the following equations.

(5) $$L_{R}(i,j)=\left\{\begin{array}{l} 1,T_{R1}<H(i,j)<T_{R2},S(i,j)>T_{S},V(i,j)>T_{V}\\ 0,else \end{array}\right.,$$

(6) $$L_{G}(i,j)=\left\{\begin{array}{l} 1,T_{G1}<H(i,j)<T_{G2},S(i,j)>T_{S},V(i,j)>T_{V}\\ 0,else \end{array}\right.,$$

(7) $$L_{B}(i,j)=\left\{\begin{array}{l} 1,T_{B1}<H(i,j)<T_{B2},S(i,j)>T_{S},V(i,j)>T_{V}\\ 0,else \end{array}\right.$$

where $L_{R}$ represents the segmentation result of the red lines, $L_{G}$ represents the segmentation result of the green lines, and $L_{B}$ represents the segmentation result of the blue lines. $T_{R1}$ denotes the low threshold and $T_{R2}$ denotes the high threshold for red line segmentation. $T_{R1}=0$ and $T_{R2}$ is denoted as the red circle in Figure 4a. $T_{G1}$ denotes the low threshold and $T_{G2}$ denotes the high threshold for green line segmentation. $T_{G1}$ is denoted as the green circle and $T_{G2}$ is denoted as the green tetragonal star in Figure 4a. $T_{B1}$ denotes the low threshold and $T_{B2}$ denotes the high threshold for blue line segmentation. $T_{B1}$ is denoted as the blue circle and $T_{B2}$ is denoted as the blue tetragonal star in Figure 4a. $T_{S}$ denotes the global threshold to separate the RGB line part from the dark part in the S channel, and it is denoted as the purple pentagram in Figure 4b. $T_{V}$ denotes the global threshold to separate the RGB line ROI from the dark background in the V channel, and it is denoted as the purple pentagram in Figure 4c. The segmented RGB lines with Equations (5)–(7) are illustrated in Figure 5. By comparing the segmented lines in Figure 5 with the acquired lines in Figure 3, it is seen that the SDD segmentation method is robust.

**Figure 6:**
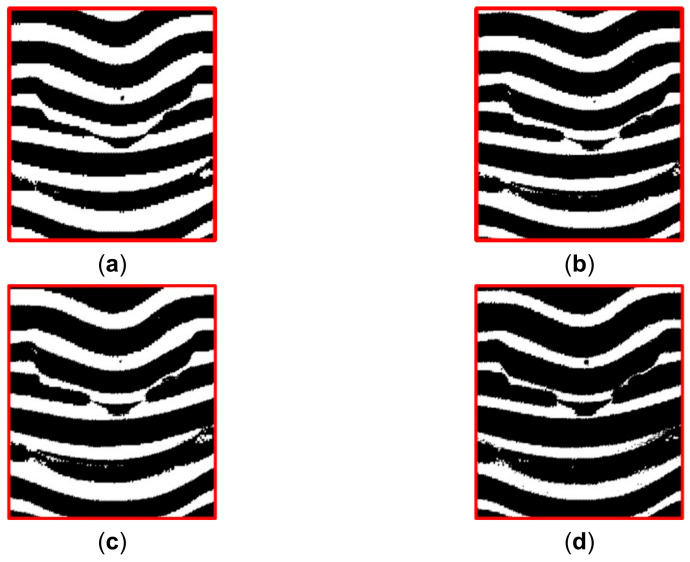
Qualitative comparison of the line segmentation results by state-of-the-art classification methods and the result by the proposed SDD method. (**a**) Result of the SDD method; (**b**) Result of K-means; (**c**) Result of Otsu; (**d**) Result of EM.

As reviewed in the second section, two existing methods [26,30] use the classification methods to segment the lines. To demonstrate the superiority of the proposed line segmentation method, the classic classification methods were tested to segment the H channel of the same illustrated image, and the line classification results were refined by the SDD segmentation results of S and V channels in the same way. The compared classic classification methods included K-means [35], Otsu’s method [36], and expectation maximization (EM) [37]. Zoomed-in parts of the classification results around the mouth and nose by different classic methods are shown in Figure 6. As can be seen, only the segmented adjacent lines by the SDD method are separate from each other. There are adjacent lines adhesive to each other in the results of the compared classic classification methods, which will make the subsequent line clustering fail.

### 3.2. The Proposed line Clustering Method

The proposed line clustering method contains the following steps.

**Step 1:** The starting line is selected based on its length and centroid in the top to bottom direction. Then, it is fitted as:

(8) $$y=a_{1}x^{7}+a_{2}x^{6}+a_{3}x^{5}+\ldots +a_{7}x+a_{8}$$

where x denotes the coordinate of the pixel on the starting line in the horizontal direction and y denotes the coordinate of the pixel on the starting line in the vertical direction. The coefficient of the fitted line is computed by the least squares error method.

(9) $${\left[a_{1},a_{2},\ldots ,a_{7},a_{8}\right]}^{T}={\left(\psi^{T}\psi \right)}^{-1}\psi^{T}Y,$$

(10) $$\psi =\;\left[\begin{array}{ccccc} x_{1}^{7} & x_{1}^{6} & \ldots & x_{1} & 1\\ x_{2}^{7} & x_{2}^{6} & \ldots & x_{2} & 1\\ \vdots & \vdots & \vdots & \vdots & \vdots \\ x_{m}^{7} & x_{m}^{6} & \ldots & x_{m} & 1 \end{array}\right],$$

(11) $$Y=\;{\left[y_{1},y_{2},\ldots ,y_{m}\right]}^{T}$$

where m denotes the total number of pixels on the starting line. The leftmost point of the starting line and the rightmost point of the starting line are computed as $\left(x_{l},y_{l}\right)$ and $\left(x_{r},y_{r}\right)$, respectively. The padded line is computed as follows.

(12) $$y=\left\{\begin{array}{l} y_{l},x<x_{l}\\ y_{r},x>x_{r} \end{array}\right..$$

**Figure 7:**
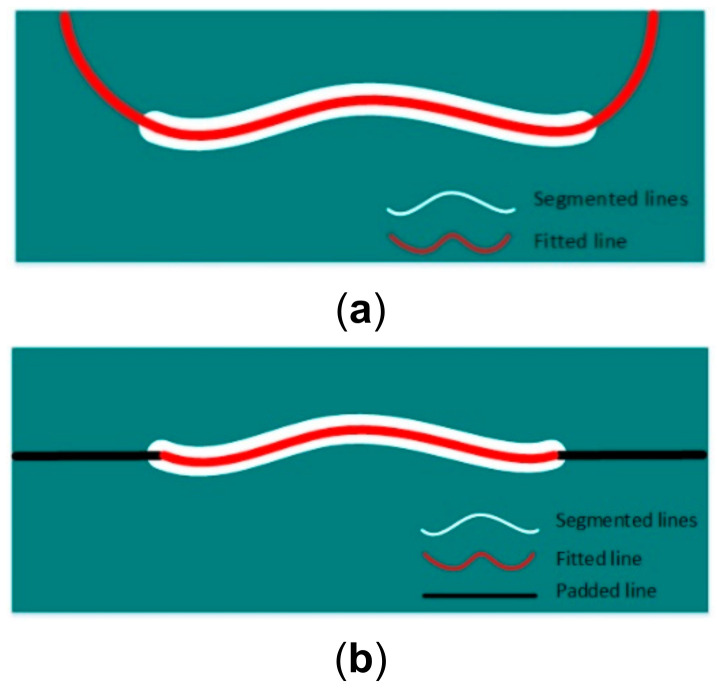
Illustration of the fitted line and the padded line. (**a**) The fitted line in red superimposed on the segmented line; (**b**) The non-overlapping part of the fitted line is cut off and replaced with the padded line.

The fitted line and the padded line are illustrated in Figure 7a,b, respectively.

**Figure 8:**
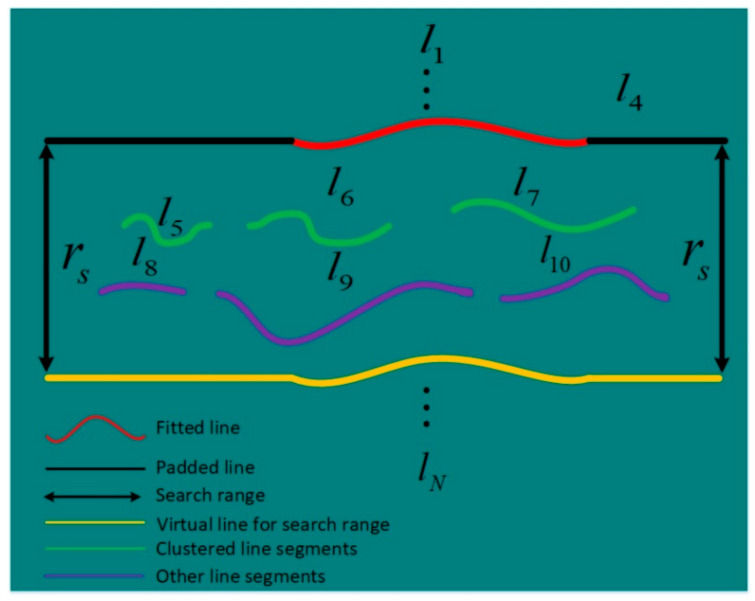
Illustration of the search range.

**Step 2:** A search range $r_{s}$ is defined based on the fitted line and the padded line. $r_{s}$ is computed as twice the average distance between adjacent lines. As illustrated in Figure 8, the search range is denoted by the virtual yellow line. All the line segments within the search rage will be selected for clustering the next line. In this illustration, the starting line is determined as the fourth segmented line. Consequently, the clustering process will include downward clustering and upward clustering.

**Step 3:** The line segments are clustered in the search range. Firstly, all the line segments in the search range are denoted as 1, i.e., all of them belong to the next clustered line by default. The exclusion method works as follows.

The centroid, the coordinate of the leftmost point, and the coordinate of the rightmost point are computed as $\left(C_{x}^{i},C_{y}^{i}\right)$, $\left(x_{l}^{i},y_{l}^{i}\right)$ and $\left(x_{r}^{i},y_{r}^{i}\right)$, respectively, for the i-th line segment $l_{i}$ in the search range. The i-th line segment $l_{i}$ is combined with any one of the remaining line segments, $l_{j}$ and j≠i. If one of the following conditions is met, the i-th line segment $l_{i}$ will be determined as not belonging to the next clustered line.

(1) If the leftmost point of the i-th line segment $l_{i}$ falls between the length range $\left[X_{l}^{j},X_{r}^{j}\right]$ of the j-th line segment $l_{j}$, i.e., $X_{l}^{i}>X_{l}^{j}$ and $X_{l}^{i}<X_{r}^{j}$, the j-th line segment $l_{j}$ is fitted with the line model by Equation (8) as $f_{j}$. If the position of the leftmost point of the i-th line segment $l_{i}$ is below the position of the fitted line model $f_{j}$ for the downward clustering, i.e., $Y_{l}^{i}-f_{j}(X_{l}^{i})>0$, the i-th line segment $l_{i}$ is determined as not belonging to the next clustered line. If the position of the leftmost point of the i-th line segment $l_{i}$ is above the position of the fitted line model $f_{j}$ for the upward clustering, i.e., $Y_{l}^{i}-f_{j}(X_{l}^{i})<0$, the i-th line segment $l_{i}$ is determined as not belonging to the next clustered line.
(2) If the rightmost point of the i-th line segment $l_{i}$ falls between the length range $\left[X_{l}^{j},X_{r}^{j}\right]$ of the j-th line segment $l_{j}$, i.e., $X_{r}^{i}>X_{l}^{j}$ and $X_{r}^{i}<X_{r}^{j}$, the j-th line segment $l_{j}$ is fitted with the line model by Equation (8) as $f_{j}$. If the position of the leftmost point of the i-th line segment $l_{i}$ is below the position of the fitted line $f_{j}$ for the downward clustering, i.e., $Y_{r}^{i}-f_{j}(X_{r}^{i})>0$, the i-th line segment $l_{i}$ is determined as not belonging to the next clustered line. If the position of the leftmost point of the i-th line segment $l_{i}$ is above the position of the fitted line $f_{j}$ for the upward clustering, i.e., $Y_{r}^{i}-f_{j}(X_{r}^{i})<0$, the i-th line segment $l_{i}$ is determined as not belonging to the next clustered line.
(3) (3) If the centroid of the i-th line segment $l_{i}$ falls between the length range $\left[X_{l}^{j},X_{r}^{j}\right]$ of the j-th line segment $l_{j}$, i.e., $C_{x}^{i}>X_{l}^{j}$ and $C_{x}^{i}<X_{r}^{j}$, the j-th line segment $l_{j}$ is fitted with the line model by Equation (8) as $f_{j}$. If the position of the leftmost point of the i-th line segment $l_{i}$ is below the position of the fitted line $f_{j}$ for the downward clustering, i.e., $C_{y}^{i}-f_{j}(C_{x}^{i})>0$, the i-th line segment $l_{i}$ is determined as not belonging to the next clustered line. If the position of the leftmost point of the i-th line segment $l_{i}$ is above the position of the fitted line $f_{j}$ for upward clustering, i.e., $C_{y}^{i}-f_{j}(C_{x}^{i})<0$, the i-th line segment $l_{i}$ is determined as not belonging to the next clustered line.
(4) If the leftmost point of the j-th line segment $l_{j}$ falls between the length range $\left[X_{l}^{i},X_{r}^{i}\right]$ of the i-th line segment $l_{i}$, i.e., $X_{l}^{j}>X_{l}^{i}$ and $X_{l}^{j}<X_{r}^{i}$, the i-th line segment $l_{i}$ is fitted with the line model by Equation (8) as $f_{i}$. If the position of the leftmost point of the j-th line segment $l_{j}$ is below the position of the fitted line $f_{i}$ for the downward clustering, i.e., $f_{i}(X_{l}^{j})-Y_{l}^{j}>0$, the i-th line segment $l_{i}$ is determined as not belonging to the next clustered line. If the position of the leftmost point of the j-th line segment $l_{j}$ is above the position of the fitted line $f_{i}$ for the upward clustering, i.e., $f_{i}(X_{l}^{j})-Y_{l}^{j}<0$, the i-th line segment $l_{i}$ is determined as not belonging to the next clustered line.
(5) If the rightmost point of the j-th line segment $l_{j}$ falls between the length range $\left[X_{l}^{i},X_{r}^{i}\right]$ of the i-th line segment $l_{i}$, i.e., $X_{r}^{j}>X_{l}^{i}$ and $X_{r}^{j}<X_{r}^{i}$, the i-th line segment $l_{i}$ is fitted with the line model by Equation (8) as $f_{i}$. If the position of the leftmost point of the j-th line segment $l_{j}$ is below the position of the fitted line $f_{i}$ for the downward clustering, i.e., $f_{i}(X_{r}^{j})-Y_{r}^{j}>0$, the i-th line segment $l_{i}$ is determined as not belonging to the next clustered line. If the position of the leftmost point of the j-th line segment $l_{j}$ is above the position of the fitted line $f_{i}$ for the upward clustering, i.e., $f_{i}(X_{r}^{j})-Y_{r}^{j}<0$, the i-th line segment $l_{i}$ is determined as not belonging to the next clustered line.
(6) If the centroid of the j-th line segment $l_{j}$ falls between the length range $\left[X_{l}^{i},X_{r}^{i}\right]$ of the i-th line segment $l_{i}$, i.e., $C_{x}^{j}>X_{l}^{i}$ and $C_{x}^{j}<X_{r}^{i}$, the i-th line segment $l_{i}$ is fitted with the line model by Equation (8) as $f_{i}$. If the position of the leftmost point of the j-th line segment $l_{j}$ is below the position of the fitted line $f_{i}$ for the downward clustering, i.e., $f_{i}(C_{x}^{j})-C_{y}^{j}>0$, the i-th line segment $l_{i}$ is determined as not belonging to the next clustered line. If the position of the leftmost point of the j-th line segment $l_{j}$ is above the position of the fitted line $f_{i}$ for the upward clustering, i.e., $f_{i}(C_{x}^{j})-C_{y}^{j}<0$, the i-th line segment $l_{i}$ is determined as not belonging to the next clustered line.

All the line segments that meet one of the above six conditions will be determined as not belonging to the next clustered line. For succinct expression and better understanding, the six conditions are formulated by the following equation.

(13) $$l_{i}=\left\{\begin{array}{cc} 0 & \left(X_{l}^{i}>X_{l}^{j}\;\&\;X_{l}^{i}<X_{r}^{j}\;\&\;Y_{l}^{i}-f_{j}(X_{l}^{i})>0\right)\\ 0 & \left(X_{r}^{i}>X_{l}^{j}\;\&\;X_{r}^{i}<X_{r}^{j}\;\&\;Y_{r}^{i}-f_{j}(X_{r}^{i})>0\right)\\ 0 & \left(C_{x}^{i}>X_{l}^{j}\;\&\;C_{x}^{i}<X_{r}^{j}\;\&\;C_{y}^{i}-f_{j}(C_{x}^{i})>0\right)\\ 0 & \left(X_{l}^{j}>X_{l}^{i}\;\&\;X_{l}^{j}<X_{r}^{i}\;\&\;f_{i}(X_{l}^{j})-Y_{l}^{j}>0\right)\\ 0 & \left(X_{r}^{j}>X_{l}^{i}\;\&\;X_{r}^{j}<X_{r}^{i}\;\&\;f_{i}(X_{r}^{j})-Y_{r}^{j}>0\right)\\ 0 & \left(C_{x}^{j}>X_{l}^{i}\;\&\;C_{x}^{j}<X_{r}^{i}\;\&\;f_{i}(C_{x}^{j})-C_{y}^{j}>0\right) \end{array}\right..$$

**Figure 9:**
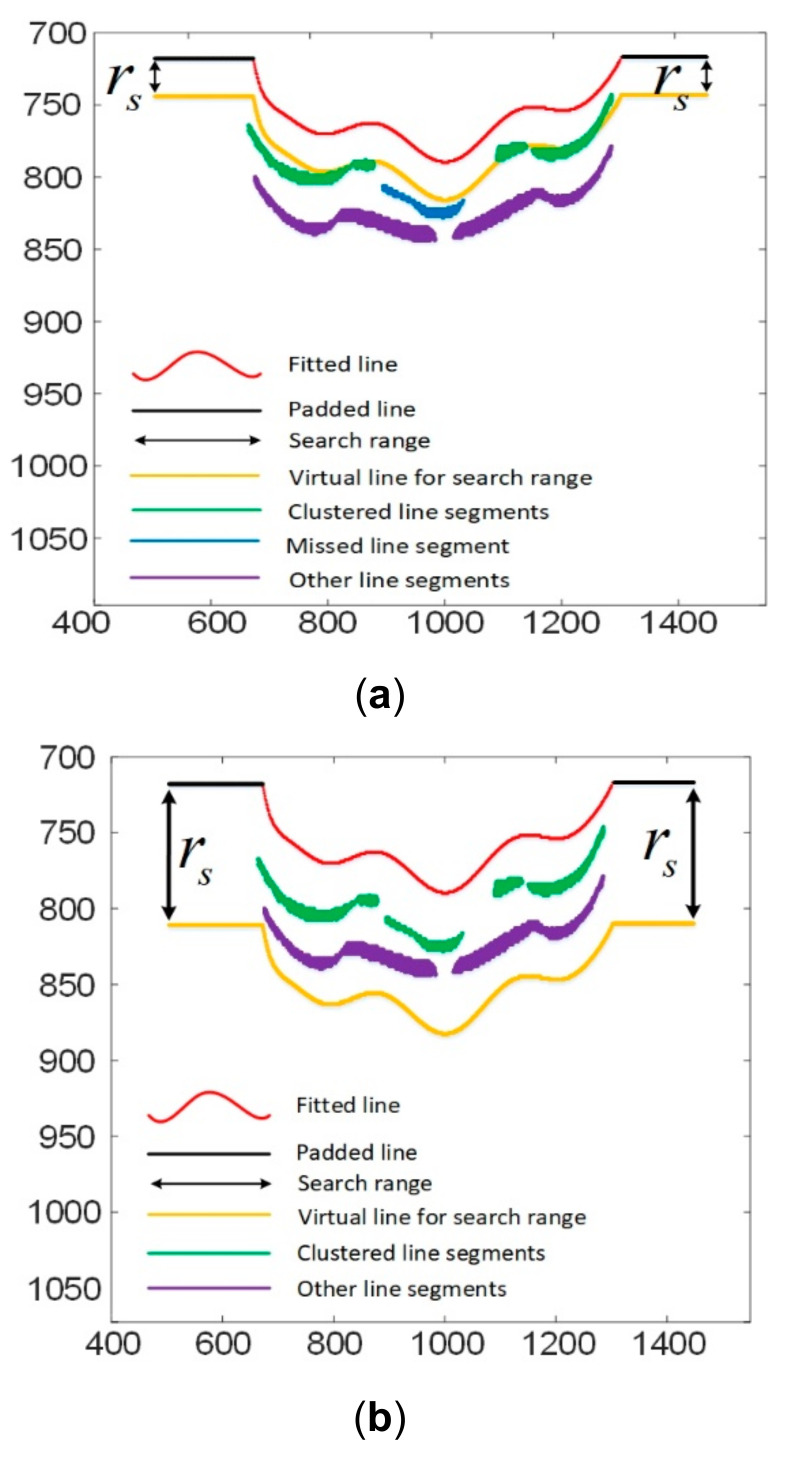
Comparison of the clustering result with different search ranges. (**a**) The clustering result with a small search range; (**b**) The clustering result with a proper search range.

As illustrated in Figure 8, the combination of the line segments $l_{5}$, $l_{6}$ and $l_{7}$ is clustered as the next line. Their combination is fitted by the line model (Equation (8)) and padded by Equation (12). Then, the newly fitted and padded line is used for the subsequent line clustering. As can be seen, the working principle of the proposed exclusion method is based on the selection of a large search range that includes line segments belonging to more than one line. If the search range is not large enough, it is easy to miss some line segments as illustrated in Figure 9.

**Step 4:** Repeat Steps 2–3 until all the lines are clustered. After all the lines are clustered, the fitted lines by Equation (8) are used to generate the phase map with the method proposed in [7].

### 3.3. Three-Dimensional Reconstruction Based on the Phase Map

**Figure 10:**
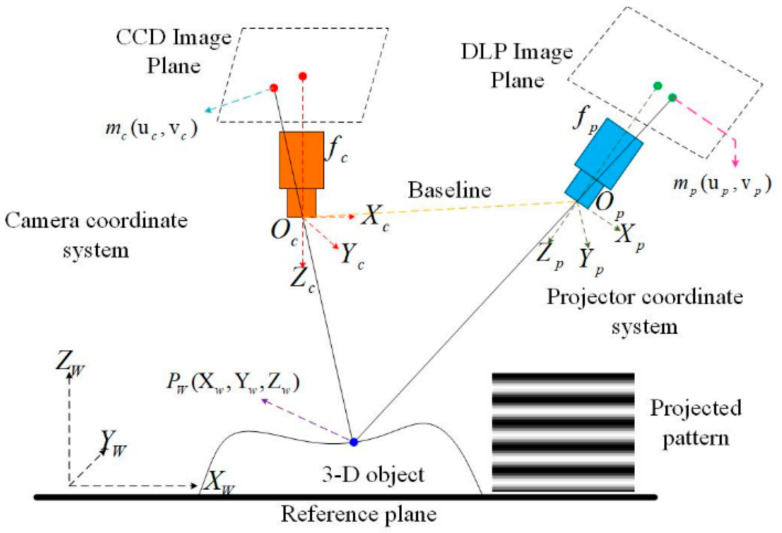
The illustration of the SL 3D imaging system.

Although the method of generating the phase map by a single line pattern was proposed in [7], its 3D reconstruction principle has not yet been explained systematically. This missing work is included in this section. As illustrated in Figure 10, the structured light 3D imaging system is composed of a projector and a camera. The camera coordinate and the world coordinate are related mathematically by the rule of the pinhole camera model [38] as follows.

(14) $$Z_{c}\left[\begin{array}{c} x_{c}\\ y_{c}\\ 1 \end{array}\right]=M_{int}M_{ext}\left[\begin{array}{c} X_{w}\\ Y_{w}\\ Z_{w}\\ 1 \end{array}\right]$$

where $\left(x_{c},y_{c}\right)$ is the camera coordinate and $\left(X_{w},Y_{w},Z_{w}\right)$ is the world coordinate. $M_{int}$ is the intrinsic matrix and $M_{ext}$ is the extrinsic matrix. They are formulated as [38]:

(15) $$M_{\mathrm{int}}=\left[\begin{array}{ccc} f_{x} & 0 & u_{c}\\ 0 & f_{y} & v_{c}\\ 0 & 0 & 1 \end{array}\right],$$

(16) $$M_{ext}=\left[\begin{array}{llll} r_{11}, & r_{12}, & r_{13}, & T_{1}\\ r_{21}, & r_{22}, & r_{23}, & T_{2}\\ r_{31}, & r_{32}, & r_{33}, & T_{3} \end{array}\right]$$

where $\left(f_{x},f_{y}\right)$ is the camera’s focal length and $\left(u_{c},v_{c}\right)$ is the camera’s principle point. $\left[r_{11},r_{12},r_{13};r_{21},r_{22},r_{23};r_{31},r_{32},r_{33}\right]$ is the rotation matrix and ${\left[T_{1},T_{2},T_{3}\right]}^{T}$ is the translation vector. For simplicity, Equation (14) can be rewritten as the following format [38].

(17) $$Z_{c}\left[\begin{array}{c} x_{c}\\ y_{c}\\ 1 \end{array}\right]=\left[\begin{array}{llll} m_{11}^{wc}, & m_{12}^{wc}, & m_{13}^{wc}, & m_{14}^{wc}\\ m_{21}^{wc}, & m_{22}^{wc}, & m_{23}^{wc}, & m_{24}^{wc}\\ m_{31}^{wc}, & m_{32}^{wc}, & m_{33}^{wc}, & m_{34}^{wc} \end{array}\right]\left[\begin{array}{c} X_{w}\\ Y_{w}\\ Z_{w}\\ 1 \end{array}\right].$$

To decrease the number of unknown parameters, we let $m_{34}^{wc}=1$. Equation (17) is then expanded into the following three equations.

(18) $$Z_{c}x_{c}=m_{11}^{wc}X_{w}+m_{12}^{wc}Y_{w}+m_{13}^{wc}Z_{w}+m_{14}^{wc},$$

(19) $$Z_{c}y_{c}=m_{21}^{wc}X_{w}+m_{22}^{wc}Y_{w}+m_{23}^{wc}Z_{w}+m_{24}^{wc},$$

(20) $$Z_{c}=m_{31}^{wc}X_{w}+m_{32}^{wc}Y_{w}+m_{33}^{wc}Z_{w}+1.$$

Substituting Equation (20) into Equation (18), the following equation is obtained.

(21) $$m_{11}^{wc}X_{w}+m_{12}^{wc}Y_{w}+m_{13}^{wc}Z_{w}+m_{14}^{wc}=x_{c}m_{31}^{wc}X_{w}+x_{c}m_{32}^{wc}Y_{w}+x_{c}m_{33}^{wc}Z_{w}+x_{c}.$$

Substituting Equation (20) into Equation (19), the following equation is obtained.

(22) $$m_{21}^{wc}X_{w}+m_{22}^{wc}Y_{w}+m_{23}^{wc}Z_{w}+m_{24}^{wc}=y_{c}m_{31}^{wc}X_{w}+y_{c}m_{32}^{wc}Y_{w}+y_{c}m_{33}^{wc}Z_{w}+y_{c}.$$

Equation (21) can be rewritten into the following format [31]:

(23) $$\left[X_{w},Y_{w},Z_{w},1,0,0,0,0,-x_{c}X_{w},-x_{c}Y_{w},-x_{c}Z_{w}\right]\theta_{c}=x_{c}$$

where

$$\theta_{c}={\left[m_{11}^{wc},m_{12}^{wc},m_{13}^{wc},m_{14}^{wc},m_{21}^{wc},m_{22}^{wc},m_{23}^{wc},m_{24}^{wc},m_{31}^{wc},m_{32}^{wc},m_{33}^{wc}\right]}^{T}.$$

In the same way, Equation (22) can be rewritten into the following format [31]:

(24) $$\left[0,0,0,0,X_{w},Y_{w},Z_{w},1,-y_{c}X_{w},-y_{c}Y_{w},-y_{c}Z_{w}\right]\theta_{c}=y_{c}.$$

Equations (23) and (24) are combined into the following equation.

(25) $$X_{c}\theta_{c}=Y_{c}$$

where $X_{c}=\left[\begin{array}{c} X_{w},Y_{w},Z_{w},1,0,0,0,0,-x_{c}X_{w},-x_{c}Y_{w},-x_{c}Z_{w}\\ 0,0,0,0,X_{w},Y_{w},Z_{w},1,-y_{c}X_{w},-y_{c}Y_{w},-y_{c}Z_{w} \end{array}\right]$ and $Y_{c}={\left[x_{c},y_{c}\right]}^{T}$.

During SL 3D system calibration, $X_{c}$ and $Y_{c}$ are known, the unknown $\theta_{c}$ can be solved by least squares method [31].

(26) $$\theta_{c}={\left(X_{c}{}^{T}X_{c}\right)}^{-1}X_{c}{}^{T}Y_{c}.$$

The mathematical relationship between the projector coordinate system and the world coordinate system also follows the rule of the pinhole camera model that is formulated as follows.

(27) $$Z_{p}\left[\begin{array}{c} x_{p}\\ y_{p}\\ 1 \end{array}\right]=\left[\begin{array}{llll} m_{11}^{wp}, & m_{12}^{wp}, & m_{13}^{wp}, & m_{14}^{wp}\\ m_{21}^{wp}, & m_{22}^{wp}, & m_{23}^{wp}, & m_{24}^{wp}\\ m_{31}^{wp}, & m_{32}^{wp}, & m_{33}^{wp}, & m_{34}^{wp} \end{array}\right]\left[\begin{array}{c} X_{w}\\ Y_{w}\\ Z_{w}\\ 1 \end{array}\right]$$

where $\left(x_{p},y_{p}\right)$ is the camera coordinate. To decrease the number of unknown parameters, we also let $m_{34}^{wp}=1$. Equation (27) is then expanded in the same way as Equation (17) into three equations. Since the projected pattern only changes in the y direction, only $y_{p}$ will be used for 3D measurement. Thus, only the following equation is used.

(28) $$\begin{array}{l} m_{21}^{wp}X_{w}+m_{22}^{wp}Y_{w}+m_{23}^{wp}Z_{w}+m_{24}^{wp}\\ =y_{p}m_{31}^{wp}X_{w}+y_{p}m_{32}^{wp}Y_{w}+y_{p}m_{33}^{wp}Z_{w}+y_{p} \end{array}$$

Equation (28) can be rewritten into the following format:

(29) $$X_{p}\theta_{p}=Y_{p}$$

where $X_{p}=\left[X_{w},Y_{w},Z_{w},1,-y_{p}X_{w},-y_{p}Y_{w},-y_{p}Z_{w}\right]$, $\theta_{p}={\left[m_{21}^{wp},m_{22}^{wp},m_{23}^{wp},m_{24}^{wp},m_{31}^{wp},m_{32}^{wp},m_{33}^{wp}\right]}^{T}$ and $Y_{p}=\left[y_{p}\right]$.

During calibration, $X_{p}$ and $Y_{p}$ are known, the unknown $\theta_{p}$ can be solved by the least squares method [31].

(30) $$\theta_{p}={\left(X_{p}{}^{T}X_{p}\right)}^{-1}X_{p}{}^{T}Y_{p}.$$

After $\theta_{c}$ and $\theta_{p}$ are obtained, they are used for 3D measurement by the following equation.

(31) $$\left[\begin{array}{c} X_{w}\\ Y_{w}\\ Z_{w} \end{array}\right]=H^{-1}\left[\begin{array}{c} x_{c}-m_{14}^{wc}\\ y_{c}-m_{24}^{wc}\\ y_{p}-m_{14}^{wp} \end{array}\right]$$

where $H=\left[\begin{array}{c} m_{11}^{wc}-x_{c}m_{31}^{wc},m_{12}^{wc}-x_{c}m_{32}^{wc},m_{13}^{wc}-x_{c}m_{33}^{wc}\\ m_{21}^{wc}-y_{c}m_{31}^{wc},m_{22}^{wc}-y_{c}m_{32}^{wc},m_{23}^{wc}-y_{c}m_{33}^{wc}\\ m_{11}^{wp}-y_{p}m_{21}^{wp},m_{12}^{wp}-y_{p}m_{22}^{wp},m_{13}^{wp}-y_{p}m_{23}^{wp} \end{array}\right]$.

The coded line pattern contains L indexed parallel lines. When the coded line pattern is projected onto the object, the parallel lines will be distorted by the depth of the object. The camera acquires the distorted pattern image with resolution M×N. The distorted lines in the acquired pattern image are clustered according to their indexes in the coded line pattern. $y_{p}$ is computed as the y coordinate of the pixel on the distorted line. The computed $y_{p}$ is stacked into an N×1 vector for each clustered line. All the vectors are then stacked into an L×N matrix. To make $y_{p}$ correspond to the $\left(x_{c},y_{c}\right)$ one by one, the L×N matrix is transformed into an M×N matrix by spline interpolation. As a result, the object can be reconstructed with the same resolution of the camera. Both the reconstruction resolution and the reconstruction accuracy can be increased when the number of indexed lines in the coded pattern is increased. On the other hand, it will be more challenging to segment and cluster dense lines.

## 4. Results and Discussion

In our established structured light 3D imaging system, a BENQ MU706 projector with the resolution 1920 × 1200 was used to project the RGB line pattern. The projector was calibrated manually as follows: (1) the projection angle of the projector with reference to the object was selected as 90°; (2) the distance between the projector and the object was adjusted to make sure that the projected pattern could cover the object completely. In the conducted experiments, the distance was chosen as 70 cm; (3) the focus of the projector was adjusted manually to make sure that the RGB lines were clearly shown on the surface of the object; (4) the contrast and brightness of the projector were adjusted manually to make sure that the RGB lines acquired by the camera were clear enough for line segmentation. A Blackfly BFLY-PGE-50A2C-CS camera with the resolution 2592 × 1944 was used to acquire the pattern images. The camera was calibrated with the MATLAB camera calibrator toolbox to calculate the distortion parameters $\left\{k_{1},k_{2},p_{1},p_{2}\right\}$ and they were calculated as $k_{1}=-0.34995$, $k_{2}=0.15753$, $p_{1}=-0.0003$, and $p_{2}=0.000377$, respectively. The structured light 3D imaging system was calibrated by a calibration grid with Equation (26) and Equation (30) to compute the parameters $\theta_{c}$ and $\theta_{p}$, respectively. The $\theta_{c}$ computed by the calibration grid with 24 known marker points was [3.7747, 0.0174, −1.0601, 974.6918, −0.0827, 3.8175, −0.8671, 383.6297, −0.0000, −0.0000, −0.0010] and the $\theta_{p}$ computed by the calibration grid with 24 known marker points was [−0.1332, 3.5434, 0.4618, 335.1717, −0.0000, −0.0002, −0.0009]. After 3D calibration, the calibration grid was used to compute the reconstruction accuracy of the structured light 3D imaging system and the computed mean root squared error (MRSE) was 0.4581 mm.

**Figure 11:**
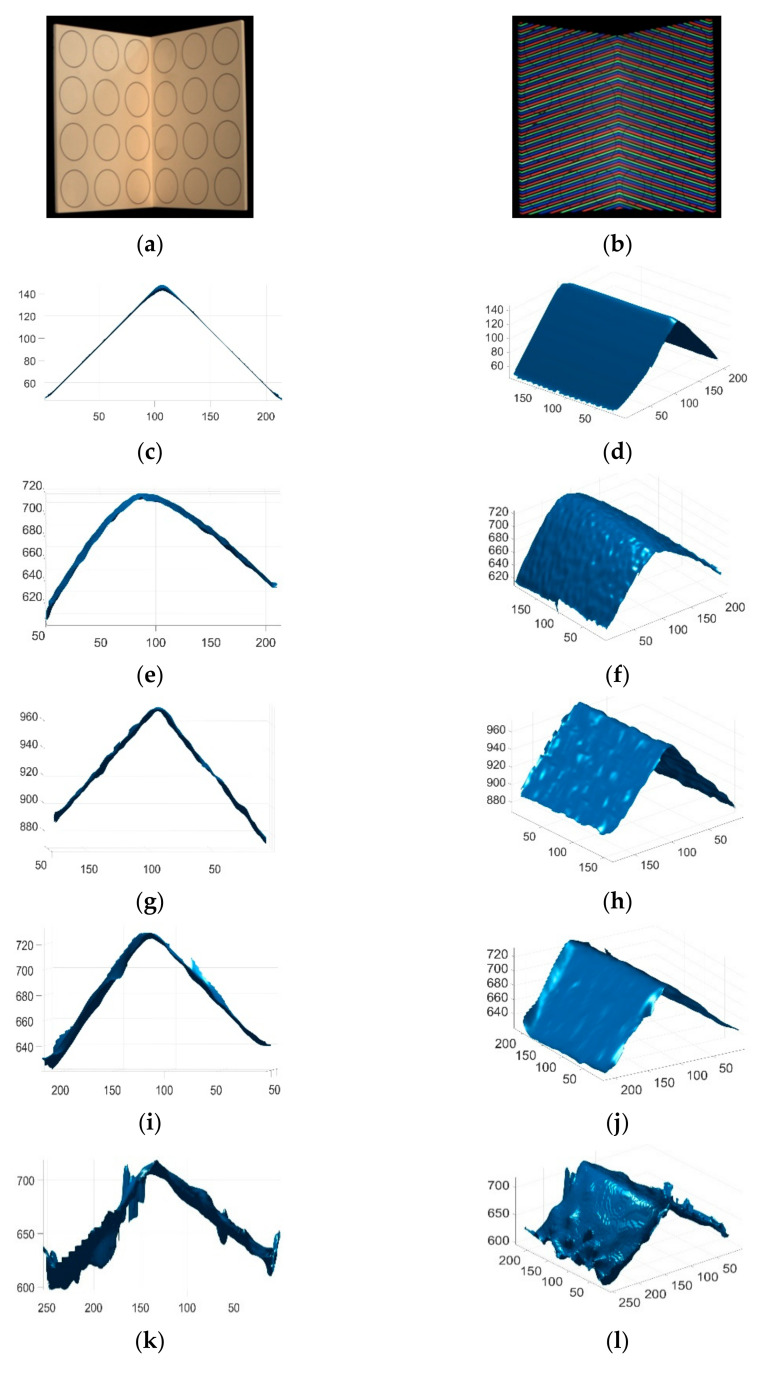
Reconstructed calibration grids by different methods. (**a**) The image of the calibration grid; (**b**) The acquired pattern image of the calibration grid; (**c**) Reconstructed calibration grid by the proposed method in view 1; (**d**) Reconstructed calibration grid by the proposed method in view 2; (**e**) Reconstructed calibration grid by Kinect V2 in view 1; (**f**) Reconstructed calibration grid by Kinect V2 in view 2; (**g**) Reconstructed calibration grid by Kinect V1 in view 1; (**h**) Reconstructed calibration grid by Kinect V1 in view 2; (**i**) Reconstructed calibration grid by Intel D435 ASV in view 1; (**j**) Reconstructed calibration grid by Intel D435 ASV in view 2; (**k**) Reconstructed calibration grid by Intel D435 SV in view 1; (**l**) Reconstructed calibration grid by Intel D435 SV in view 2.

**Figure 12:**
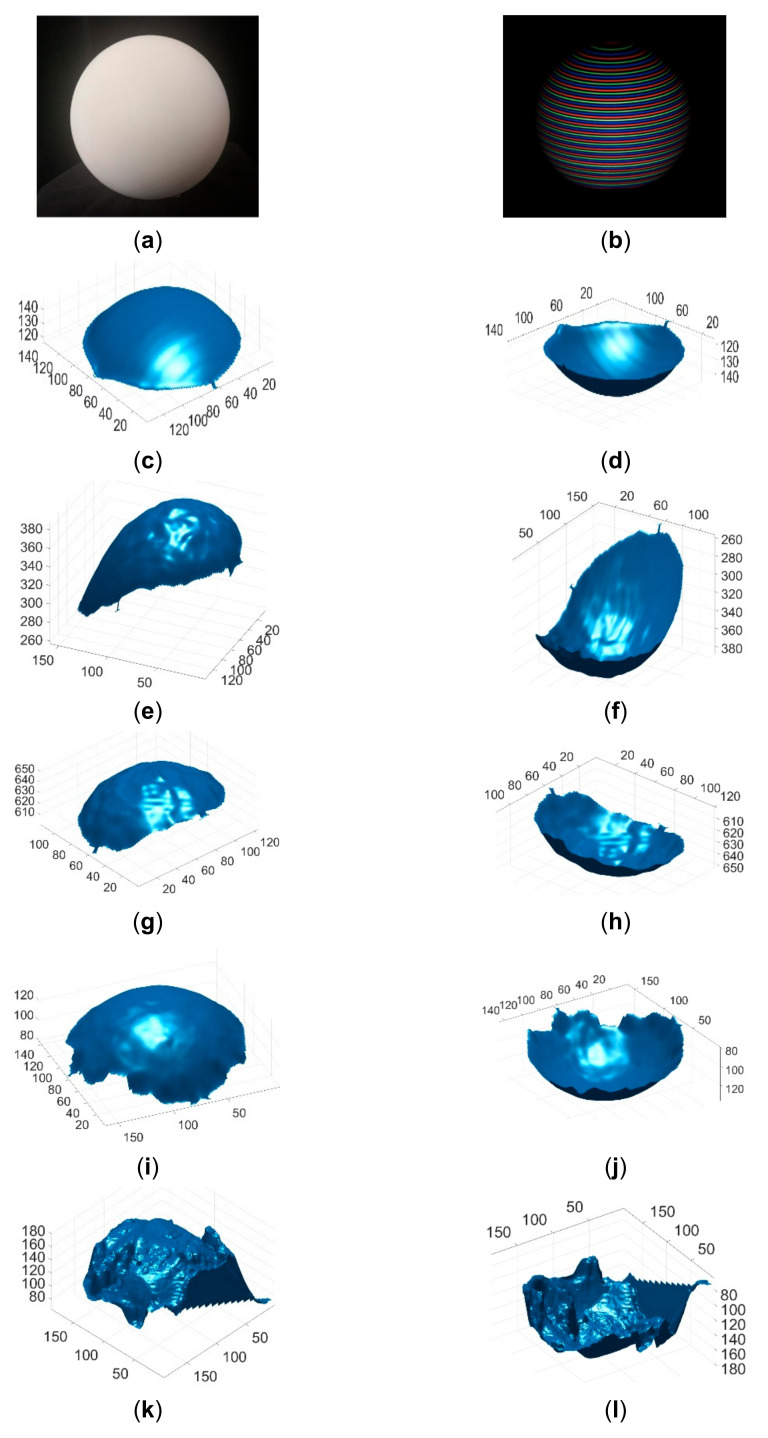
Reconstructed balls by different methods. (**a**) The image of the ball; (**b**) The acquired pattern image of the ball; (**c**) Reconstructed ball by the proposed method in view 1; (**d**) Reconstructed ball by the proposed method in view 2; (**e**) Reconstructed ball by Kinect V2 in view 1; (**f**) Reconstructed ball by Kinect V2 in view 2; (**g**) Reconstructed ball by Kinect V1 in view 1; (**h**) Reconstructed ball by Kinect V1 in view 2; (**i**) Reconstructed ball by Intel D435 ASV in view 1; (**j**) Reconstructed ball by Intel D435 ASV in view 2; (**k**) Reconstructed ball by Intel D435 SV in view 1; (**l**) Reconstructed ball by Intel D435 SV in view 2.

The proposed method was compared with state-of-the-art single-shot 3D surface imaging methods (products) in measuring both static and moving objects. The compared methods included: (1) passive stereo vision (Intel realsense D435 SV); (2) active stereo vision (Intel RealSense D435 ASV); (3) light coding (Kinect V1); and (4) time of flight (Kinect V2). To compare the reconstruction accuracy quantitatively, these single-shot imaging systems were used to reconstruct the calibration grid and a ball. The reconstruction error was evaluated with MRSE, and the reconstruction errors of the different methods are compared in Table 2. As can be seen, the accuracy of the proposed method was significantly better than those of existing methods. The reconstructed calibration grids and the reconstructed balls of different methods are shown in Figure 11 and Figure 12, respectively. As can be seen, the reconstruction results of the existing methods contained significantly more noise. In addition, there were obvious distortions in the reconstruction results of existing methods. The angle between the two planes of the calibration grid was 90°, and the proposed method restored the angle much better than the other methods.

**Figure 13:**
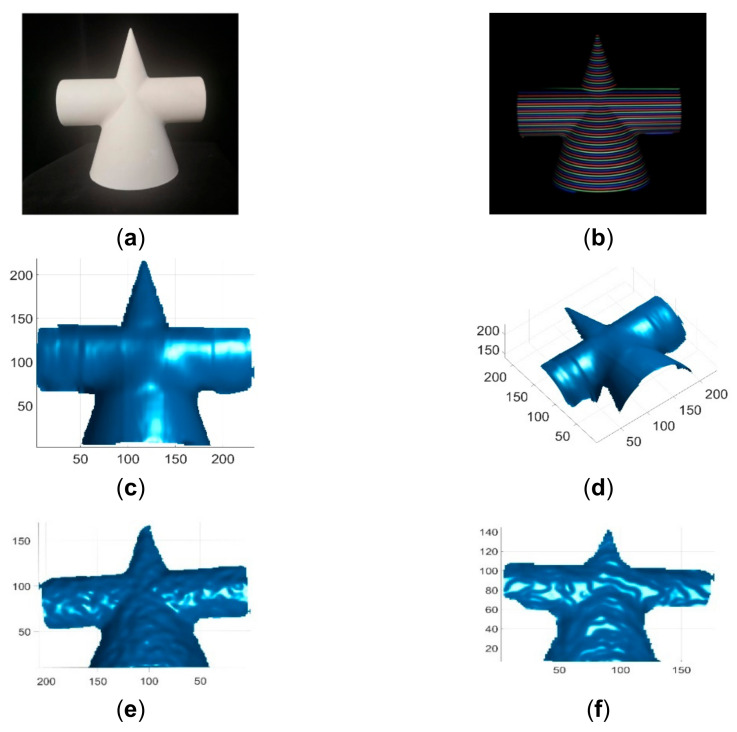

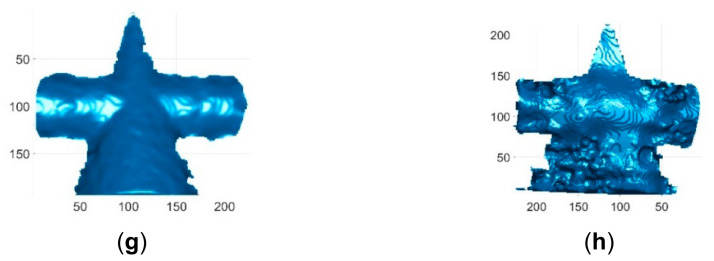
Reconstruction results of a cone with cylinder. (**a**) The cone with cylinder image; (**b**) The acquired pattern image of the cone with cylinder; (**c**) Reconstructed cone with cylinder by the proposed method in view 1; (**d**) Reconstructed cone with cylinder by the proposed method in view 2; (**e**) Reconstructed cone with cylinder by Kinect V2; (**f**) Reconstructed cone with cylinder by Kinect V1; (**g**) Reconstructed cone with cylinder by Intel D435 ASV; (**h**) Reconstructed cone with cylinder by Intel D435 SV.

**Figure 14:**
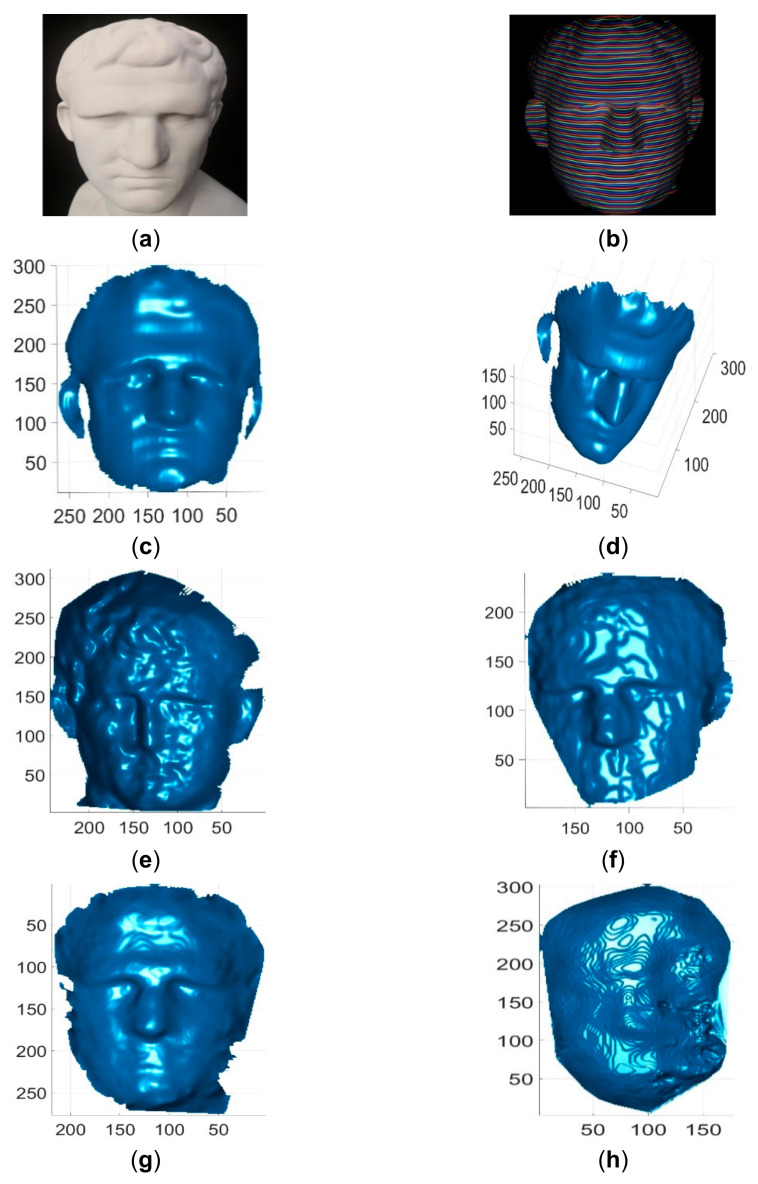
Reconstruction results of a face statue. (**a**) The face statue image; (**b**) The acquired pattern image of the face statue; (**c**) Reconstructed face statue by the proposed method in view 1; (**d**) Reconstructed face statue by the proposed method in view 2; (**e**) Reconstructed face statue by Kinect V2; (**f**) Reconstructed face statue by Kinect V1; (**g**) Reconstructed face statue by Intel D435 ASV; (**h**) Reconstructed face statue by Intel D435 SV.

**Figure 15:**
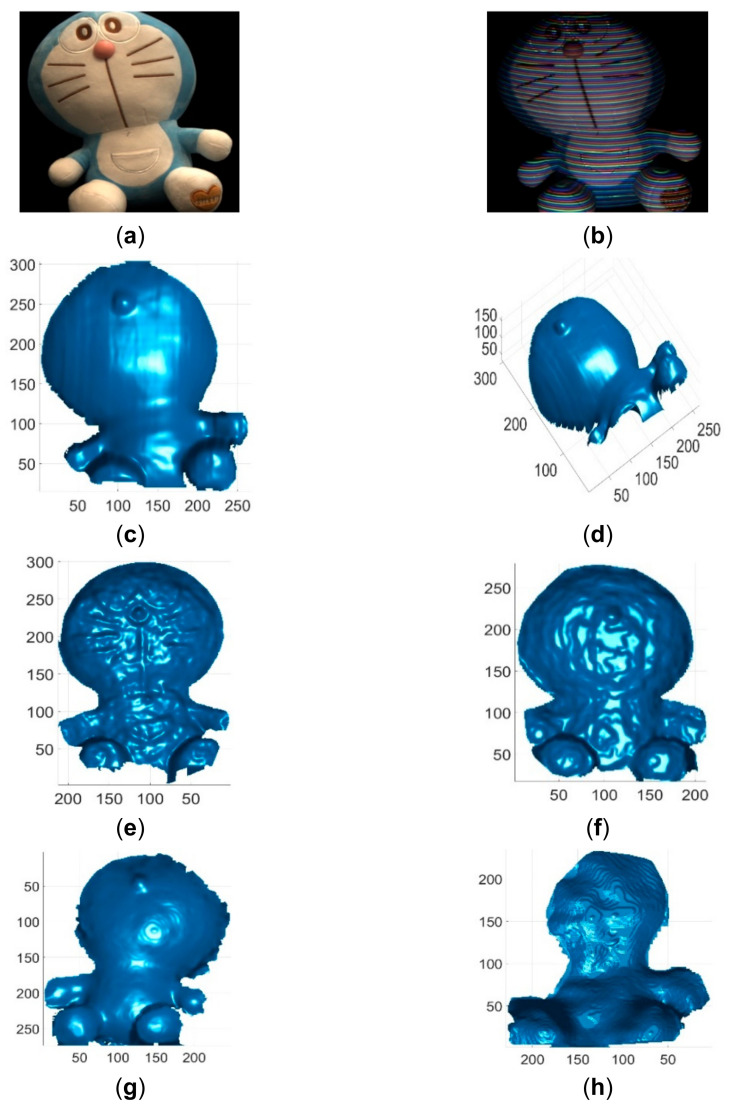
Reconstruction results of a colorful toy. (**a**) The toy image; (**b**) The acquired pattern image; (**c**) Reconstructed colorful toy by the proposed method in view 1; (**d**) Reconstructed colorful toy by the proposed method in view 2; (**e**) Reconstructed colorful toy by Kinect V2; (**f**) Reconstructed colorful toy by Kinect V1; (**g**) Reconstructed colorful toy by Intel D435 ASV; (**h**) Reconstructed colorful toy by Intel D435 SV.

**Figure 16:**
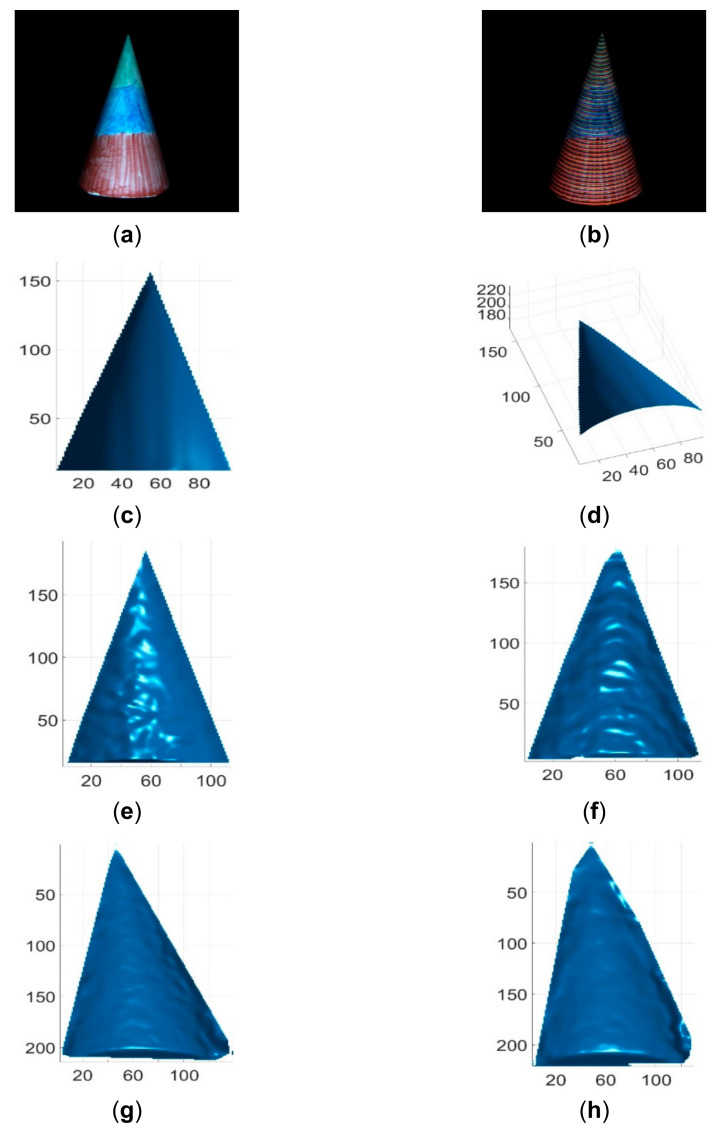
Reconstruction results of a cone with RGB color. (**a**) The RGB cone image; (**b**) The acquired pattern image of the cone; (**c**) Reconstructed cone by the proposed method in view 1; (**d**) Reconstructed cone by the proposed method in view 2; (**e**) Reconstructed cone by Kinect V2; (**f**) Reconstructed cone by Kinect V1; (**g**) Reconstructed cone by Intel D435 ASV; (**h**) Reconstructed cone by Intel D435 SV.

**Figure 17:**
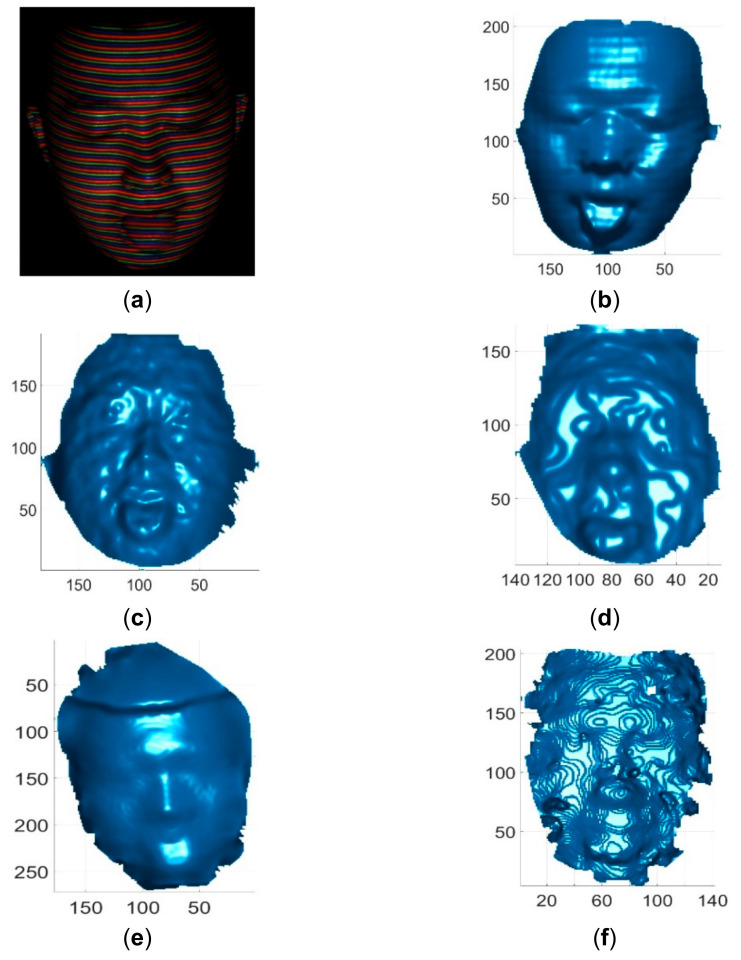
Reconstruction results of a deforming human face. (**a**) The acquired pattern image of the human face; (**b**) Reconstructed human face by the proposed method; (**c**) Reconstructed human face by Kinect V2; (**d**) Reconstructed human face by Kinect V1; (**e**) Reconstructed human face by Intel D435 ASV; (**f**) Reconstructed human face by Intel D435 SV.

These single-shot imaging systems were used to reconstruct two colorless and static objects for qualitative comparions. One colorless object was a simple cone with cylinder, and its reconstruction results by different methods are shown in Figure 13. The other colorless object was a complex face statue, and its reconstruction results by different methods are shown in Figure 14. As can be seen, the reconstruction results of the proposed method looked better than those of existing methods. In addition, these imaging sytems were used to reconstruct three static objects with colors. The reconstruction results are shown in Figure 15 and Figure 16, respectively. As can be seen, the reconstruction results of the proposed method also looked better than those of the existing methods. Finally, these single-shot imaging systems were used to recontruct deforming and moving objects. The typical results of reconstructing a deforming face by different methods are shown in Figure 17, and more reconstruction results of deforming objects by different methods are available in the attached videos (Deforming face and Deforming hand). From all the reconstruction results by different methods, it is seen that the proposed method is significantly more accurate than state-of-the-art single-shot 3D imaging methods.

**Figure 18:**
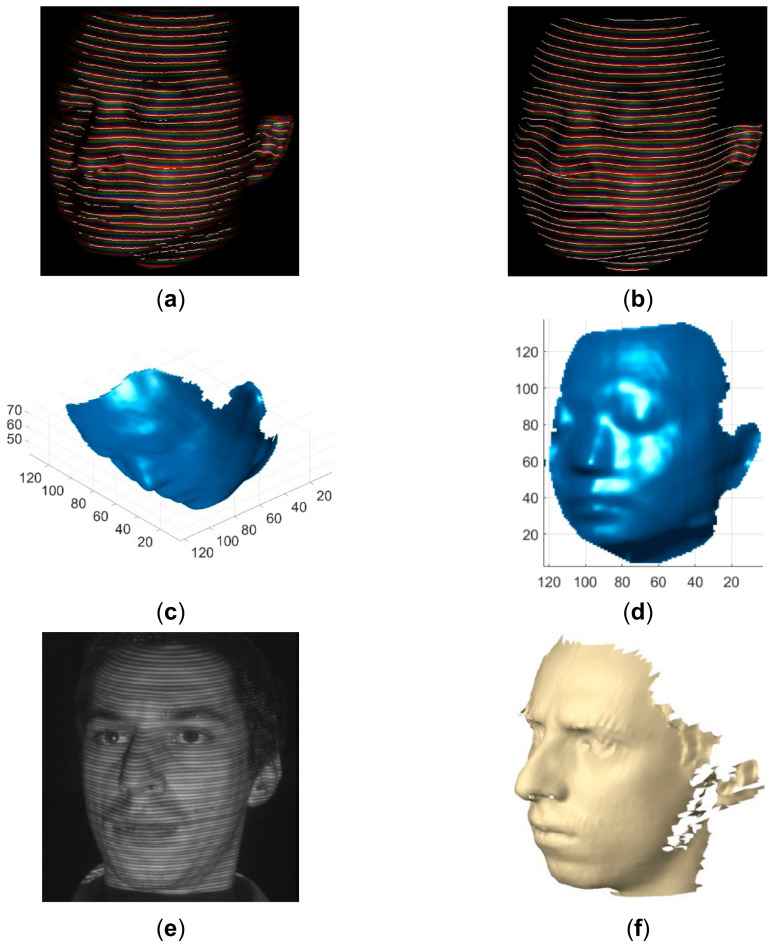
Qualitative comparison of the proposed method with the method proposed in [28]. (**a**) The detected red lines by local maxima superimposed on the acquired pattern image; (**b**) The extracted red lines by the proposed method superimposed on the acquired pattern image; (**c**) The reconstructed human face by the proposed method in view 1; (**d**) The reconstructed human face by the proposed method in view 2; (**e**) The acquired pattern image in [28]; (**f**) The reconstructed human face by the method in [28].

**Figure 19:**
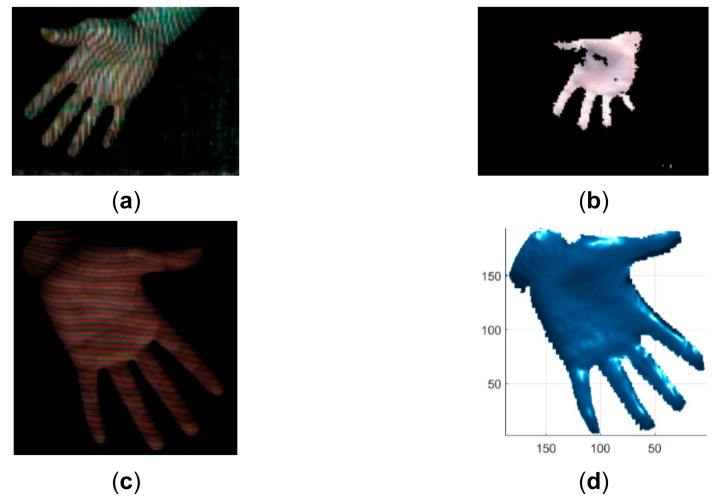
Qualitative comparison of the proposed method with the method proposed in [30]. (**a**) The acquired pattern image in [30]; (**b**) The reconstructed hand in [30]; (**c**) The acquired pattern image in this study; (**d**) The reconstructed hand by the proposed method.

**Figure 20:**
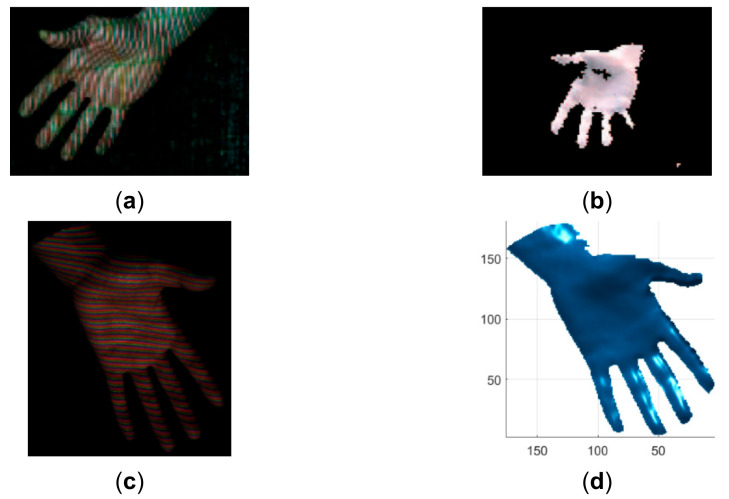
Qualitative comparison of the proposed method with the method proposed in [30]. (**a**) The acquired pattern image in [30]; (**b**) The reconstructed hand in [30]; (**c**) The acquired pattern image in this study; (**d**) The reconstructed hand by the proposed method.

**Figure 21:**
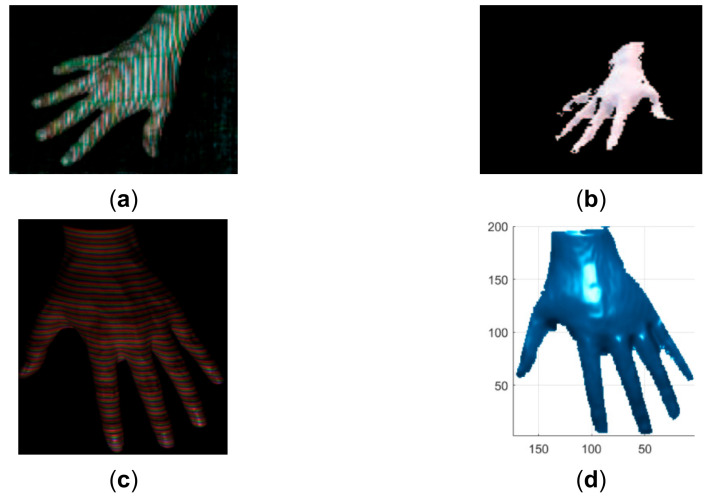
Qualitative comparison of the proposed method with the method proposed in [30]. (**a**) The acquired pattern image in [30]; (**b**) The reconstructed hand in [30]; (**c**) The acquired pattern image in this study; (**d**) The reconstructed hand by the proposed method.

**Figure 22:**
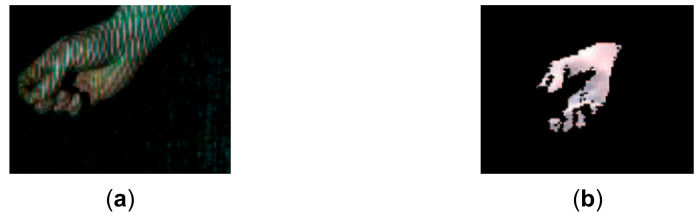

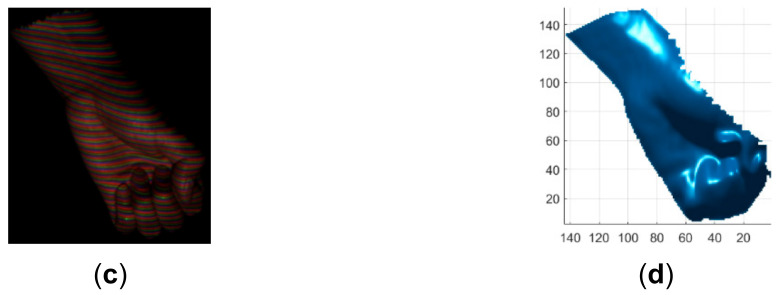
Qualitative comparison of the proposed method with the method proposed in [30]. (**a**) The acquired pattern image in [30]; (**b**) The reconstructed hand in [30]; (**c**) The acquired pattern image in this study; (**d**) The reconstructed hand by the proposed method.

**Figure 23:**
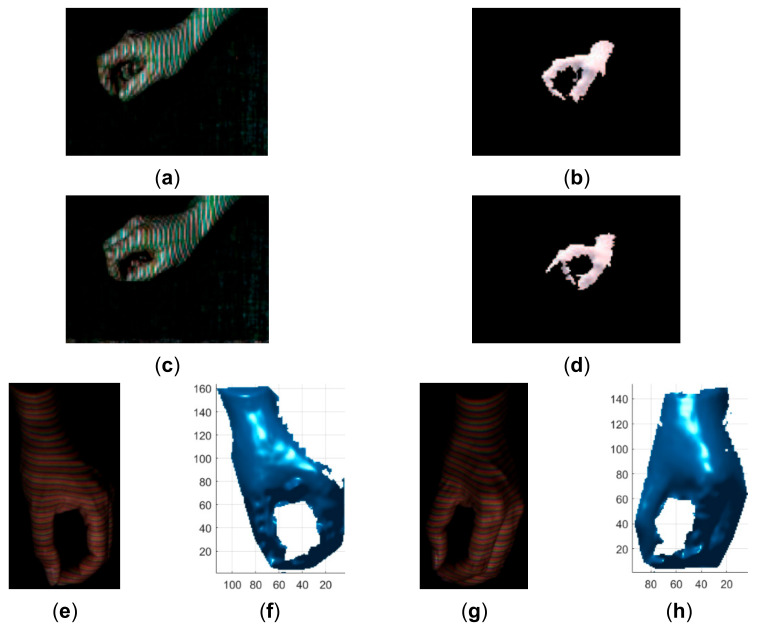
Qualitative comparison of the proposed method with the method proposed in [30]. (**a**) The acquired pattern image in [30]; (**b**) The reconstructed hand in [30]; (**c**) The acquired pattern image in [30]; (**d**) The reconstructed hand in [30]; (**e**) The acquired pattern image in this study; (**f**) The reconstructed hand by the proposed method; (**g**) The acquired pattern image in this study; (**h**) The reconstructed hand by the proposed method.

To verify the advantage of the proposed method over existing line indexing methods [28,30], the results of reconstructing similar objects are compared in Figure 18, Figure 19, Figure 20, Figure 21, Figure 22 and Figure 23. The red lines detected by local maxima [28] are shown in Figure 18a and the red lines extracted by the proposed method are shown in Figure 18b. It is seen that the lines extracted by the proposed method are more accurate and more complete. In addition, the lines detected by local maxima could not be clustered robustly by the method proposed in this paper and the methods proposed previously in [7,8,9]. By comparing the reconstructed face by the proposed method in Figure 18c,d and the reconstructed face from [28] in Figure 18f, it is seen that the ear part reconstructed by the proposed method is significantly better. Similar to [30], a moving and deforming hand was reconstructed by the proposed method and the reconstructed hands were compared with those with similar gestures from [30] in Figure 19, Figure 20, Figure 21, Figure 22 and Figure 23. As can be seen, the reconstruction results of the proposed method are more accurate and more complete than the reconstruction results from [30].

As verified by the experimental results, the usability of the proposed method includes profile measurement, 3D motion reconstruction and other related or similar applications. To use the proposed method correctly and to reduce the measurement uncertainties, users must know the limitations of the proposed method as follows.

(1) The measurement distance between the object and the structured light 3D imaging system is constrained in a relatively small range to make sure that both the camera and the projector are in focus, which is a common limitation of structured light technology.
(2) The proposed method is not suitable for applications that require the reconstruction results to be full of accurate 3D details, such as defect detection.

## 5. Conclusions

In conclusion, a RGB line-pattern-based single-shot structured light method is proposed in this paper. The proposed method addresses the challenges of line segmentation and line indexing more effectively and systematically than existing methods [26,27,28,29,30]. The RGB lines are segmented by SDD multiple-threshold selection in the HSV color space, which is significantly more accurate than classic classification methods. The guaranteed line segmentation accuracy makes the subsequent line indexing feasible. In this paper, a more robust line clustering method is proposed to index the segmented lines by an exclusion scheme, which is more effective than existing methods in dealing with the occlusions and large discontinuities. The proposed single-shot structured light method was verified by extensive experimental results of reconstructing a deforming face and a deforming hand. In addition, the accuracy of the proposed approach in measuring a calibration grid was 0.46 mm, which is significantly better than the second-best accuracy, 0.79 mm achieved by Intel D435 ASV. The accuracy of the proposed approach in measuring a ball was 0.24 mm, which is significantly better than the second-best accuracy, 0.36 mm achieved by Kinect V2.

## Figures and Tables

**Table 1:** Comparison of the major differences between the proposed method and the existing methods [26,27,28,29,30].

Methods	Line Segmentation	Line Indexing
[26]	Color classification	Permutation
[27]	Local maxima	Flood fill
[28]	Local maxima	Spanning tree
[29]	Manual	Unique word
[30]	Color classification	Graph cut
Proposed	SDD thresholds	Exclusion

**Table 2:** Quantitative accuracy comparison of the proposed method with existing single-shot methods (products) (The bold values denote the best results).

Methods\Objects	Grid	Ball
Intel D435 SV	5.83 mm	5.3 mm
Intel D435 ASV	0.79 mm	0.86 mm
Kinect V1	14.36 mm	1.18 mm
Kinect V2	3.05 mm	0.36 mm
**Proposed**	**0.46 mm**	**0.24 mm**

## Data Availability

Not applicable.

## References

[1] Wang Z.Z. (2020). Review of real-time threee-dimensional shape measurement techniques. Measurement.

[2] Al Ismaeil K., Aouada D., Solignac T., Mirbach B., Ottersten B. (2016). Real-Time Enhancement of Dynamic Depth Videos with Non-Rigid Deformations. IEEE Trans. Pattern Anal. Mach. Intell..

[3] Sabater N., Almansa A., Morel J.-M. (2011). Meaningful Matches in Stereovision. IEEE Trans. Pattern Anal. Mach. Intell..

[4] Jepsen G. (2018). Projectors for Intel^®^ RealSense™ Depth Cameras D4xx. Intel Support.

[5] Ulrich L., Vezzetti E., Moos S., Marcolin F. (2020). Analysis of RGB-D camera technologies for supporting different facial usage scenarios. Multimed. Tools Appl..

[6] Takeda M., Motoh K. (1983). Fourier transform profilometry for the automatic measurement of 3-D object shapes. Appl. Opt..

[7] Wang Z., Yang Y. (2018). Single-shot three-dimensional reconstruction based on structured light line pattern. Opt. Lasers Eng..

[8] Wang Z.Z. (2017). Unsupervised Recognition and Characterization of the Reflected Laser Lines for Robotic Gas Metal Arc Welding. IEEE Trans. Ind. Inform..

[9] Wang Z.Z. (2020). Robust three-dimensional face reconstruction by one-shot structured light line pattern. Opt. Lasers Eng..

[10] Beumier C., Acheroy M. (1998). Automatic Face Authentication from 3D surface. Br. Mach. Vis. Conf..

[11] Kemelmacher-Shlizerman I., Basri R. (2011). 3D Face Reconstruction from a Single Image Using a Single Reference Face Shape. IEEE Trans. Pattern Anal. Mach. Intell..

[12] Garrido P., Valgaerts L., Wu C., Theobalt C. (2013). Reconstructing detailed dynamic face geometry from monocular video. ACM Trans. Graph..

[13] Moss J., Linney A., Grindrod S., Mosse C. (1989). A laser scanning system for the measurement of facial surface morphology. Opt. Lasers Eng..

[14] You Y., Shen Y., Zhang G., Xing X. (2017). Real-Time and High-Resolution 3D Face Measurement via a Smart Active Optical Sensor. Sensors.

[15] Vilchez-Rojas H.L., Rayas J.A., Martínez-García A. (2020). Use of white light profiles for the contouring of objects. Opt. Lasers Eng..

[16] Chen S.Y., Li Y.F., Zhang J. (2008). Vision Processing for Realtime 3-D Data Acquisition Based on Coded Structured Light. IEEE Trans. Image Process..

[17] Payeur P., Desjardins D. (2009). Structured Light Stereoscopic Imaging with Dynamic Pseudo-random Patterns. Image Analysis and Recognition, ICIAR 2009.

[18] Griffin P.M., Narasimhan L.S., Yee S.R. (1992). Generation of uniquely encoded light patterns for range data acquisition. Pattern Recognit..

[19] Morano R., Ozturk C., Conn R., Dubin S., Zietz S., Nissano J. (1998). Structured light using pseudorandom codes. IEEE Trans. Pattern Anal. Mach. Intell..

[20] Ito M., Ishii A. (1995). A three-level checkerboard pattern (TCP) projection method for curved surface measurement. Pattern Recognit..

[21] Vuylsteke P., Oosterlinck A. (1990). Range image acquisition with a single binary-encoded light pattern. IEEE Trans. Pattern Anal. Mach. Intell..

[22] Wang J., Xiong Z., Wang Z., Zhang Y., Wu F. (2015). FPGA Design and Implementation of Kinect-Like Depth Sensing. IEEE Trans. Circuits Syst. Video Technol..

[23] Khoshelham K., Elberink S.O. (2012). Accuracy and Resolution of Kinect Depth Data for Indoor Mapping Applications. Sensors.

[24] Zhang Y., Xiong Z., Yang Z., Wu F. (2013). Real-Time Scalable Depth Sensing with Hybrid Structured Light Illumination. IEEE Trans. Image Process..

[25] Li W., Li H., Zhang H. (2020). Light plane calibration and accuracy analysis for multi-line structured light vision measurement system. Optik.

[26] Je C., Lee S.W., Park R.-H. (2012). Colour-stripe permutation pattern for rapid structured-light range imaging. Opt. Commun..

[27] Robinson A., Alboul L., Rodrigues M. (2004). Methods for indexing stripes in uncoded structured light scanning systems. J. WSCG.

[28] Brink W., Robinson A., Rodrigues M.A. (2008). Indexing Uncoded Stripe Patterns in Structured Light Systems by Maximum Spanning Trees. BMVC.

[29] Boyer K.L., Kak A.C. (1987). Color-Encoded Structured Light for Rapid Active Ranging. IEEE Trans. Pattern Anal. Mach. Intell..

[30] Koninckx T., Van Gool L. (2006). Real-time range acquisition by adaptive structured light. IEEE Trans. Pattern Anal. Mach. Intell..

[31] Yalla V.G., Hassebrook L.G. (2005). Very high resolution 3D surface scanning using multi-frequency phase measuring profilometry. Def. Secur..

[32] Wang Z. (2014). Robust measurement of the diffuse surface by phase shift profilometry. J. Opt..

[33] Wang Z. (2020). Robust segmentation of the colour image by fusing the SDD clustering results from different colour spaces. IET Image Process..

[34] Wang Z. (2021). Automatic Localization and Segmentation of the Ventricles in Magnetic Resonance Images. IEEE Trans. Circuits Syst. Video Technol..

[35] Lloyd S. (1982). Least squares quantization in PCM. IEEE Trans. Inf. Theory.

[36] Otsu N. (1979). A threshold selection method from gray-level histograms. IEEE Trans. Syst. Man Cybern..

[37] Dempster A.P., Laird N.M., Rubin D.B. (1977). Maximum likelihood from incomplete data via the EM algorithm. J. R. Stat. Soc..

[38] Hartley R., Zisserman A., Faugeras O. (2004). Multiple View Geometry in Computer Vision.

